# RNAi-based Boolean gates in the yeast *Saccharomyces cerevisiae*

**DOI:** 10.3389/fbioe.2024.1392967

**Published:** 2024-06-04

**Authors:** Ximing Tian, Andrey Volkovinskiy, Mario Andrea Marchisio

**Affiliations:** ^1^ School of Pharmaceutical Science and Technology, Tianjin University, Tianjin, China; ^2^ School of Life Science and Technology, Harbin Institute of Technology, Harbin, China

**Keywords:** siRNA precursor, Boolean gates, convergent promoters, antisense transcription, *Saccharomyces cerevisiae*

## Abstract

Boolean gates, the fundamental components of digital circuits, have been widely investigated in synthetic biology because they permit the fabrication of biosensors and facilitate biocomputing. This study was conducted to design and construct Boolean gates in the yeast *Saccharomyces cerevisiae*, the main component of which was the RNA interference pathway (RNAi) that is naturally absent from the budding yeast cells. We tested different expression cassettes for the siRNA precursor (a giant hairpin sequence, a DNA fragment—flanked by one or two introns—between convergent promoters or transcribed separately in the sense and antisense directions) and placed different components under the control of the circuit inputs (i.e., the siRNA precursor or proteins such as the Dicer and the Argonaute). We found that RNAi-based logic gates are highly sensitive to promoter leakage and, for this reason, challenging to implement *in vivo*. Convergent-promoter architecture turned out to be the most reliable solution, even though the overall best performance was achieved with the most difficult design based on the siRNA precursor as a giant hairpin.

## Introduction

Small interfering RNA (siRNA) molecules are noncoding RNAs that play a significant role in the regulation of gene expression (Lam et al., 2015) at the translational level in eukaryotic cells. siRNAs are fully complementary to the transcript of the target genes and are silenced through a mechanism known as RNA interference (RNAi) (Agrawal et al., 2003). Initially, a siRNA precursor, which is usually a double-stranded RNA (dsRNA) molecule up to 100 nt long, is cleaved in the cytoplasm into smaller (21–23-nt long (Zamore et al., 2000)) pieces (the siRNAs) by the Dicer (Dcr) enzyme. siRNAs then become part of the RNA-induced silencing complex (RISC), where their passenger (sense) strand is removed. The guide (antisense) strand, in contrast, directs the RISC to the target mRNA (Sontheimer, 2005) that is finally cut by the Argonaute (Ago) endonuclease and is then degraded by the cell. Due to their ability to silence specific genes, siRNAs have attracted significant attention as potent instruments for targeted drug delivery and disease treatment (Semizarov et al., 2003).

The yeast *Saccharomyces cerevisiae*, a commonly used organism in molecular biological research and biotechnology applications (Blount et al., 2012), does not naturally express a complete RNAi pathway (Feldmann, 2011). However, upon introduction of *Dcr* and *Ago* in the yeast genome, RNA interference can be re-established (Wang et al., 2006; Drinnenberg et al., 2009) and employed in synthetic gene circuits to downregulate protein expression at the mRNA level as an alternative to ribozymes/riboswitches (Babiskin and Smolke, 2011; Groher et al., 2018; Liu et al., 2023), PUF proteins (JACKSON et al., 2004), and CRISPR-(d)Cas systems (d: DNase-deficient) (Borchardt et al., 2015; Zhang et al., 2022; Yu and Marchisio, 2023).

Drinnenberg and colleagues (Drinnenberg et al., 2009) were the first to show how to re-engineer RNAi in *S. cerevisiae*. They implemented a circuit where the siRNA precursor was synthesized, as a giant hairpin, under the *GAL1* promoter (pGAL1) that is activated by galactose and repressed by glucose. siRNA molecules, which were generated by the action of Dcr1 and Ago1 from *Saccharomyces castelli* on the siRNA precursor, targeted a region of the green fluorescent protein (GFP) transcript. Ideally, galactose (input: 1) would trigger the RNAi pathway that determines the degradation of the GFP mRNA, causing a low fluorescence output (0). Glucose (input: 0), in contrast, would stop RNAi and determine a high GFP expression (output: 1). Therefore, the circuit should behave like a logical “NOT” gate. However, the circuit always exhibited low fluorescence both with and without galactose in the cell culture. Later research focusing on RNA interference in *S. cerevisiae* did not consider chemically inducible/repressible or protein-regulated promoters to express the siRNA precursor or any other RNAi components. The constitutive promoters of different strengths were used inside the original circuit by Drinnenberg and colleagues (Drinnenberg et al., 2009; Crook et al., 2014). Alternatively, novel strategies to generate the siRNA precursor (such as convergent promoters or separate sense–antisense gene expression—Si et al., 2015; Crook et al., 2016) were adopted. Hence, RNAi was never employed again to realize Boolean gates or more complex digital circuits in yeast.

Digital circuits have emerged as a significant branch of synthetic biology, finding widespread application in medical diagnosis, environmental care, and biocomputing (Benenson, 2012; Cheng and Lu, 2012). *S. cerevisiae* cells have been turned into Boolean gates, mainly by using orthogonal bacterial transcription factors such as TetR (Bellí et al., 1998; Murphy et al., 2007), LacI (Mazumder and McMillen, 2014), LexA (Rantasalo et al., 2018), and the CRISPR-dCas9 system (Farzadfard et al., 2013). At the translation level, ribozymes (Win and Smolke, 2008) and riboswitches (Kötter et al., 2009) have been engineered to respond to specific input signals such as theophylline and tetracycline. Moreover, a consortia of yeast cells exchanging pheromones have been shown to mimic what are effectively logic gates sensing up to three inputs (Regot et al., 2011). Logic circuits provide a clear, compact representation of the relationship between their inputs (usually chemical species) and output (e.g., fluorescence) in a truth table which contains only 0/1 values and from which two Boolean formulae (one in the conjunctive, the other in the disjunctive normal form) are derived (Marchisio and Stelling, 2011). Inputs can be categorized into inducer and corepressor groups. Inducers promote transcription by inactivating repressors or activating activators, whereas corepressors prevent RNA synthesis by activating repressors or inactivating activators (Lewin et al., 2011).

In this study, we assessed the feasibility of constructing Boolean gates that sense one or two chemicals by harnessing RNAi in *S. cerevisiae*. First, we tried to elucidate why the original circuit by Drinnenberg and colleagues failed to reproduce a NOT logic function. We then determined how to modify it and build a working NOT gate. Moreover, we constructed other Boolean gates (“YES” and “IMPLY”) based on inducible convergent promoters and separate sense–antisense transcription. The comparison of the performance of the different circuit designs led us to conclude that the convergent-promoter architecture is the most reliable solution for assembling RNAi-based digital circuits in budding yeast.

## Results and discussion

### Reconstructing the first *S. cerevisiae* synthetic gene circuit based on a reengineered RNAi pathway

**FIGURE 1:**
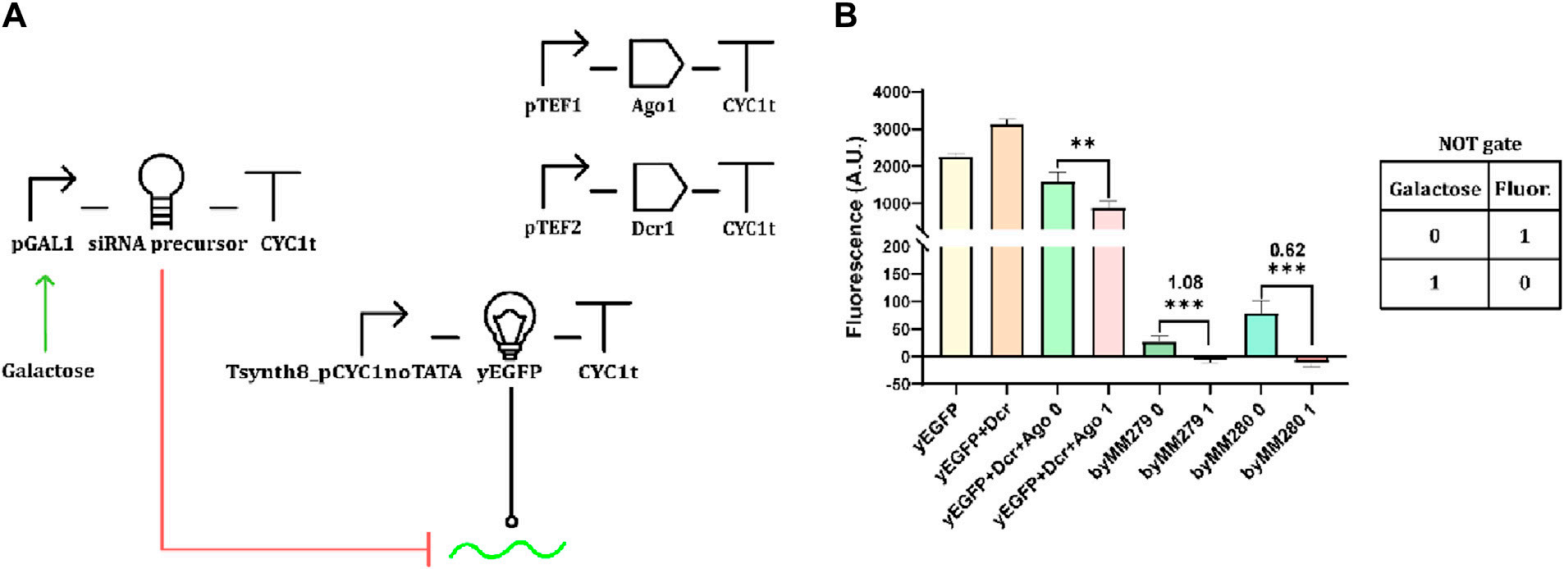
Our version of the RNAi-based circuit presented by Drinnenberg and colleagues (Drinnenberg et al., 2009). **(A)** Circuit diagram. The red hammer-shaped line represents inhibition of translation (here due to mRNA degradation); the green arrow indicates transcription activation. The black line ending with a circle indicates DNA transcription, and the green wavy line denotes the mRNA of the yEGFP (all symbols are summarized in Supplementary Figure S1). **(B)** Performance evaluation. Fluorescence was measured via flow cytometry (as from every other circuit assembled in this work—see “Materials and Methods”) from two different strains (byMM279 and byMM280) hosting the complete circuit and three other strains containing just one (yEGFP), two (yEGFP + Dcr1), and three (yEGFP + Dcr1+Ago1) TUs (the last is regarded as the control circuit). The background fluorescence (corresponding to the original, non-engineered yeast strain byMM584) was subtracted from all fluorescence levels in the bar plot, which explains why some fluorescence levels are negative. However, the OFF/ON ratios reported in the figure were calculated by including the background fluorescence to avoid negative quotients. byMM279 failed to reproduce a NOT gate because the decrease in fluorescence due to galactose induction was lower than that in the control circuit when cultured in SDC supplied with 2% galactose, which determined an OFF/ON ratio even higher than 1 (see Eq. 2 in Materials and Methods). byMM280 returned a better logic behavior. However, the OFF/ON ratio overcame the working threshold (0.5, which represents a two-fold decrease—Abraha and Marchisio, 2022). Mean fluorescence and standard deviation were computed on at least three independent experiments performed on different days (**: *p*-value <0.01; ***: *p*-value <0.001; two-sided Welch’s t-test, see Supplementary Table S2).

The starting point of our work was to build a gene circuit as close as possible to that of Drinnenberg and colleagues (Drinnenberg et al., 2009). Four transcription units (TUs) were assembled (Figure 1A). pGAL1 controlled the expression of the siRNA precursor that was designed as a giant hairpin, with a stem comprising 276 nt, whereas 67 nt were present in the loop. The siRNA molecules generated from the siRNA precursor targeted the yeast-enhanced green fluorescent protein (yEGFP) transcript (Sheff and Thorn, 2004). As in our reference work (Wang et al., 2006), Ago1 and Dcr1 were placed downstream of the *TEF1* (pTEF1) and *TEF2* promoter (pTEF2), respectively. Unlike the original circuit (Wang et al., 2006), we used a relatively weak synthetic promoter (designed in our lab) termed “Tsynth8_pCYC1noTATA” (Song et al., 2016) to lead yEGFP expression (see Supplementary Table S1 for a comparison of the strengths the promoters used in this work).

As shown in Figure 1B, we reproduced the same trend as in Drinnenberg et al. (2009)—RNAi determined a remarkable decrease in the fluorescent output both in the presence and absence of galactose.

Drinnenberg and colleagues proposed two possible explanations for this unexpected result: 1) the presence of a constitutive antisense promoter downstream of the siRNA precursor sequence; 2) pGAL1 leakage—that is, its transcriptional activity in the presence of glucose. Before proceeding with the construction of new logic circuits, we conducted a series of experiments to understand what hindered the reproduction of a NOT logic function via the synthetic RNAi pathway.

To force the termination of the transcription started by a possible antisense promoter on chromosome V (where we integrated the TU expressing the siRNA precursor), we inserted the strong *DEG1* terminator (DEG1t) (Brambilla et al., 1997) on the antisense strand just downstream of the *CYC1* terminator (CYC1t). This resulted in a new extended TU referred to as pGAL1-siRNA_precursor-CYC1t-DEG1t’ (where the prime symbol indicates that DEG1t lies on the antisense strand—Supplementary Figure S2A). However, the fluorescence expressed in the presence of glucose remained very low, not even statistically significantly different from that induced by galactose (Supplementary Figure S2B, C; Supplementary Table S3).

We performed a different test by placing pGAL1-siRNA_ precursor-CYC1t on the centromeric vector pRSII416 (Chee and Haase, 2012), which contains only the *S. cerevisiae URA3* promoter that is oriented as pGAL1 (Supplementary Figure S3A). Thus, in this new design, there should not be any antisense production of the siRNA precursor. However, as a possible drawback, a centromeric plasmid can be taken up by the yeast cells in more than a single copy (up to three) (Sikorski and Hieter, 1989). Therefore, on average, the amount of the siRNA precursor (in the presence of both glucose and galactose) is higher than in the original circuit based fully on plasmid integration. As shown in Supplementary Figure S3B and C (and Supplementary Table S4), cells growing in glucose-supplied medium were again characterized by a dramatic drop in green fluorescence expression.

Our previous tests were performed despite the fact that the *CYC1* terminator is classified as bidirectional (Uwimana et al., 2017). As such, it should be a transcription-end signal for RNA polymerase II moving in both directions along the DNA. However, in previous work from our lab (Song et al., 2016), we showed that the efficiency element of a terminator behaves as a TATA box and fosters DNA transcription if a TSS (transcription start site) lies 40–120 nucleotides downstream. In order to exclude the source of the antisense transcription being CYC1t itself, we conducted a further test where we replaced CYC1t with a poly(T) sequence (Han et al., 2023) as a transcription-termination signal (Supplementary Figure S4A). However, this modification also failed to prevent a considerable drop in the fluorescence signal in the presence of glucose in the cell cultures (Supplementary Figure S4B, C; Supplementary Table S5).

On the basis of these results, we can exclude the production of the siRNA precursor by pGAL1 in the OFF state being due to the activity of an antisense promoter. In the absence of other plausible explanations, we conclude that pGAL1 leakage is responsible for a non-negligible synthesis of the siRNA precursor in a glucose-containing cell-growth solution which fully activates the RNAi pathway. According to our fluorescence measurement and mRNA quantification (Supplementary Table S6), pGAL1 leakage in the presence of glucose corresponds to less than 0.5% of its activity in a 2%-galactose-supplied medium. Whenever we used pGAL1 to express proteins in different kinds of synthetic gene circuits, its leakage always turned out to be insignificant for the circuit’s functionality (Li et al., 2018; Yu and Marchisio, 2021; Abraha and Marchisio, 2022; Liu et al., 2023; Yu and Marchisio, 2023). Hence, promoter leakage might be a major hurdle to constructing gene digital circuits based on RNA interference.

### A new circuit design: inducible expression of the Dicer enzyme

**FIGURE 2:**
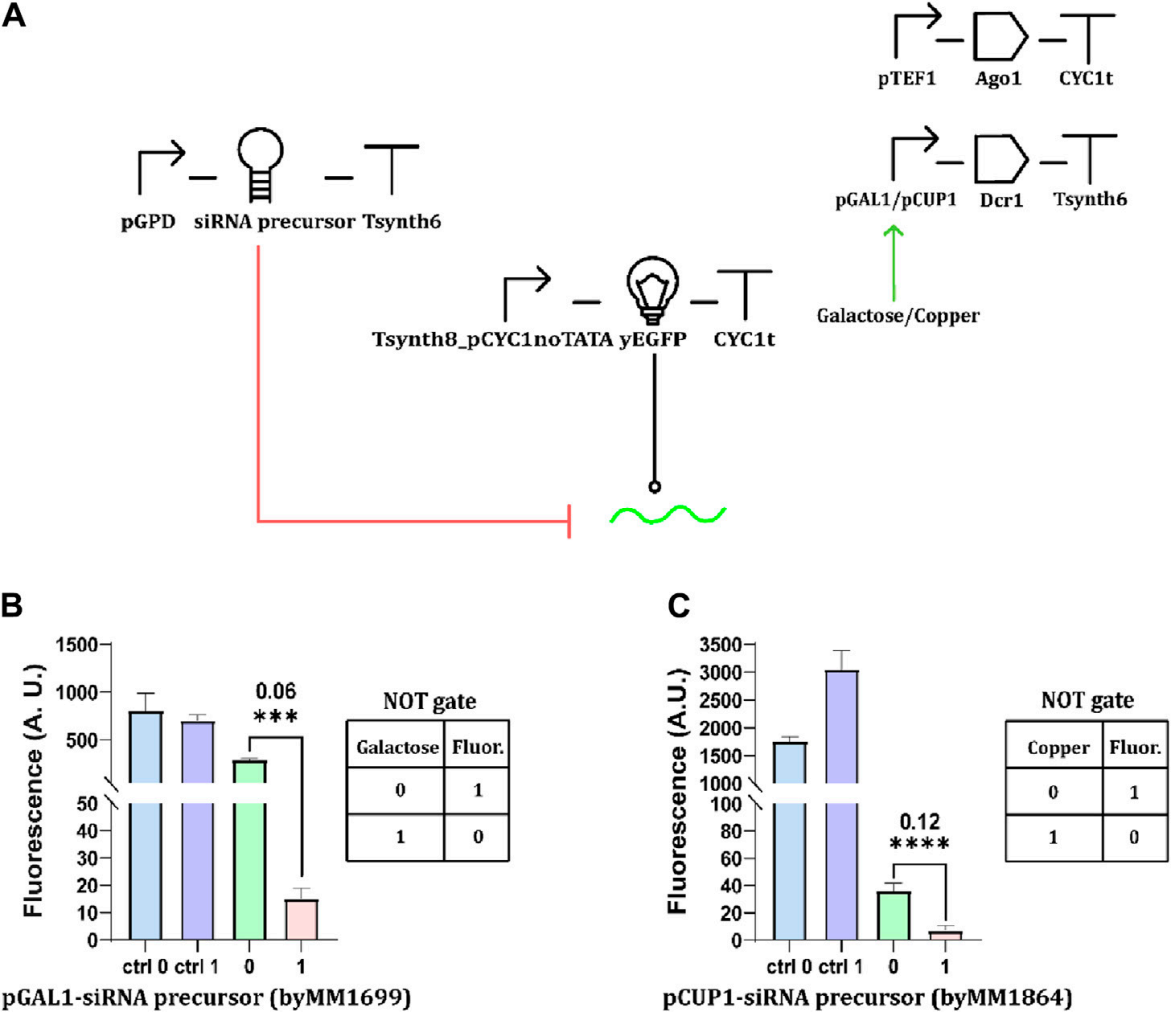
Inducible synthesis of Dcr1. **(A)** Schematic representation of NOT gates sensing either galactose or CuSO_4_. pGAL1 or pCUP1 regulates the expression of the Dcr1 protein. The siRNA precursor is constitutively expressed under pGPD. **(B)** Fluorescence intensity profile of galactose-responsive NOT gate. Cell cultures were supplied with 2% galactose. **(C)** Performance of copper-sensing NOT gate. The “1” concentration of CuSO_4_ corresponded to 0.5 mM (*: *p*-value <0.05, **: *p*-value <0.01, ***: *p*-value <0.001, ****: *p*-value <0.0001; two-sided Welch’s t-test, see Supplementary Table S7).

Moving from the observations we made on *GAL1* promoter leakage, we designed an alternative version of the circuit in Figure 1A, where the siRNA precursor was constitutively transcribed under the strong *GPD* promoter (pGPD), whereas Dcr1 expression became galactose-inducible (Figure 2A). In the presence of glucose, the limited number of Dcr1 proteins that were produced did not provoke any dramatic reduction in fluorescence expression with respect to the control circuit (where the Dcr1-expressing cassette was missing). In contrast, the fluorescence signal dropped remarkably in the presence of galactose (Figure 2B). With an OFF/ON ratio of 0.06, the circuit behaved very efficiently as a NOT gate. In order to build more basic Boolean gates that are responsive to different inputs, we first replaced pGAL1 with the copper-inducible *CUP1* promoter (Shetty et al., 2004). The resulting NOT gate still showed a low OFF/ON ratio (0.12). However, pCUP1 leakage (Supplementary Table S6) spoilt the “1” fluorescence output considerably lower than that of the control circuit (Figure 2C). Finally, we designed a YES (buffer) gate by placing Dcr1 downstream of the *MET25* repressible promoter, where pMET25 is only active in the absence of methionine (Møller et al., 2017). At high concentrations of the amino acid (i.e., 10 mM), the promoter is considerably but not completely repressed (Supplementary Table S6), preventing the realization of a properly working YES gate (Supplementary Figures S5; Supplementary Table S8). Taken together, basic YES/NOT Boolean gates based on RNAi are quite sensitive to promoter leakage, more than those realized as fully transcriptional networks (Marchisio, 2014). An inducible promoter never leads to the synthesis of a long siRNA precursor, such as the giant hairpin, since even a very low leakage can destroy the logic behavior completely. Moreover, inducible/repressible promoters associated with higher leakage activity can provoke a failure in the logic gate’s working also when driving the production of a protein, such as the Dicer enzyme. Perhaps only a careful balance of the strength of all (constitutive and non) promoters involved in an RNAi-based gene logic gate might limit the negative effects of the leakage on circuit functionality.

### Building a two-input IMPLY gate

**FIGURE 3:**
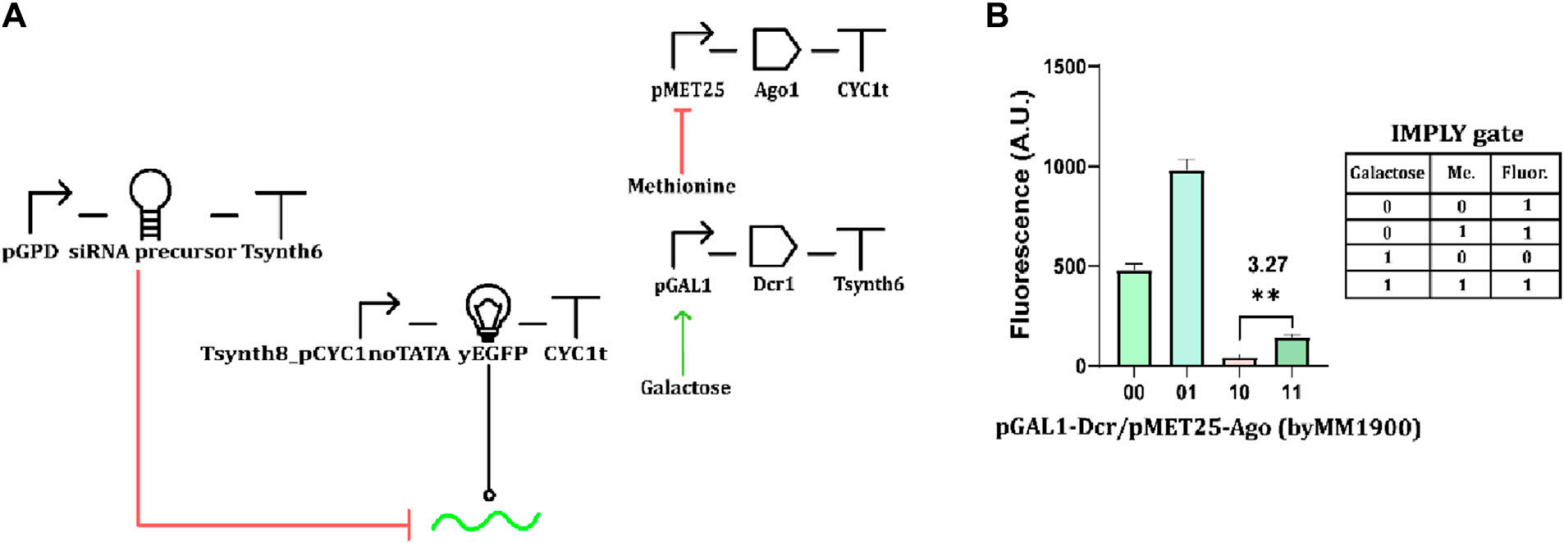
RNAi-based IMPLY gate. **(A)** Circuit scheme. The two inputs are methionine and galactose. The former represses the expression of Ago1, and the latter enhances that of Dcr1. **(B)** Fluorescence levels from the strain byMM 1900, which hosts the IMPLY gate, by varying the concentration of the two inputs (“1” concentration of methionine: 10 mM; “1” galactose amount: 2%). The minimal “1” and the only “0” output are statistically significantly different (**: *p*-value <0.01; two-sided Welch’s t-test—see Supplementary Table S9) yielding ρ = 3.27.

In previous work from our lab (Yu and Marchisio, 2023), we implemented two versions of an IMPLY gate based on mRNA degradation, hosting both pCUP1 and pMET25. The mRNA cleavage was due to the action of type V dCas12a proteins on their direct repeat (previously added to the target transcript), which was not affected by the leakage of the two promoters. Thus, we tried to determine whether the combination of pGAL1 or pCUP1 with pMET25 would permit engineering an IMPLY gate based on RNAi (the importance of this kind of gate is explained in Regot et al., 2011). In our first attempt, we kept Dcr1 under pCUP1 and replaced pTEF1 with pMET25 for the Ago1 synthesis. The circuit, however, failed to mimic the IMPLY logic behavior (Supplementary Figure S6; Supplementary Table S9). We then replaced pCUP1 with the stronger and less leaky pGAL1 (Figure 3A). Even though the “1” output obtained in the presence of both chemicals was considerably lower than the other two “1” fluorescence levels, the circuit ρ value reached 3.27 (Figure 3B and Eq. 3 in Materials and Methods), well above the working threshold ( ρ = 2).

### Convergent promoters to generate dsRNA molecules

In the previous circuits, the siRNA precursor was synthesized as a giant hairpin. A different strategy to generate siRNA-precursor-like structures—double-stranded RNAs of different sizes—relies on convergent promoter architecture (Drinnenberg et al., 2009; Si et al., 2015; Bordoy et al., 2016; Crook et al., 2016). Here, an approximately 200-nt-long DNA sequence (Si et al., 2015) corresponding to a piece of the target gene is placed between two promoters oriented in opposite directions. Sense and antisense RNA molecules of different lengths are then generated because of transcriptional interference (Shearwin et al., 2005). They bind along complementary regions and form dsRNAs that activate the RNAi pathway after being recognized and processed by the Dicer enzyme. We applied convergent promoters to the construction of the same basic Boolean gates (YES, NOT, and IMPLY) described above to assess whether this architecture could lead to better and more stable results with respect to the logic circuits expressing a giant hairpin structure.

### Testing transcriptional interference in *S. cerevisiae*: protein production

**FIGURE 4:**
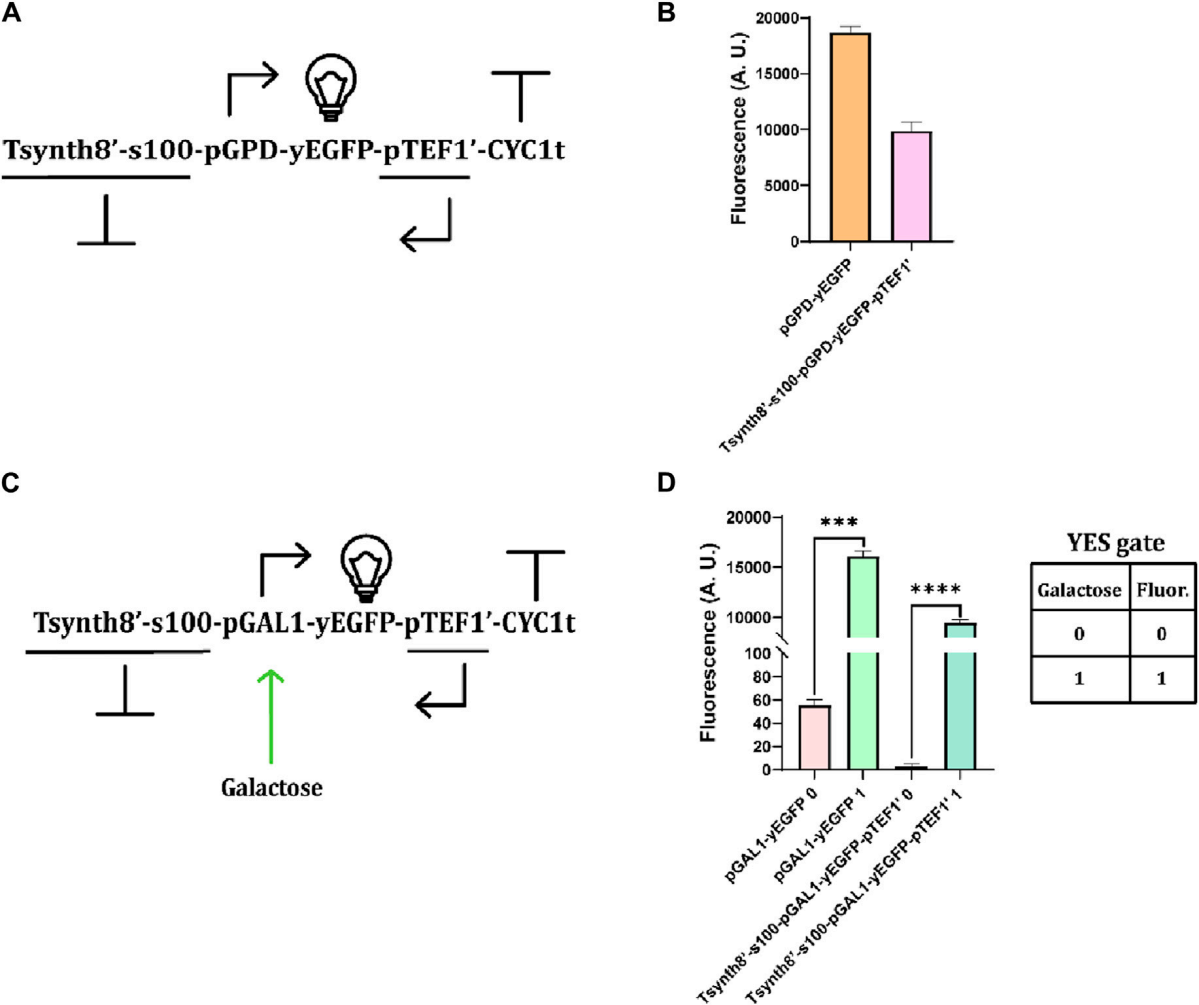
Preliminary tests on convergent promoters. **(A)** Schematic representation of the uninduced TU containing convergent promoters. The 100-nt-long spacer (spacer100, denoted as s100) was placed between Tsynth8’ (the reverse synthetic Tsynth8 terminator—Curran et al., 2015) and pGPD to avoid a possible decrease in the transcription initiation rate (Song et al., 2016). **(B)** Comparison of fluorescence intensity from a single sense promoter and a convergent promoter system. Transcriptional interference caused a near 47% decrease in fluorescence expression (Supplementary Table S10). **(C)** Galactose-inducible convergent-promoter system. **(D)** Comparison of fluorescence levels expressed in the absence and presence of galactose by a unidirectional TU and the convergent-promoter system. The drop in fluorescence, upon full induction, is over 41%. (***: *p*-value< 0.001, ****: *p*-value< 0.0001; two-sided Welch’s t-test—see also Supplementary Table S10).

We conducted a preliminary investigation on the effects of transcriptional interference due to convergent promoters on the expression of a reporter protein. In the initial test, we used two constitutive promoters (pGPD on the sense strand and pTEF1 on the antisense strand) to express the yEGFP (Figure 4A). Compared to a strain yeast containing the only sense TU (i.e., pGPD-yEGFP-CYC1t), the convergent promoter design caused a significant 46.98% decrease in the fluorescence level (Figure 4B). We then replaced pGPD with pGAL1 (realizing, in this way, a YES Boolean gate—see Figure 4C). The comparison with a single-directed TU (pGAL1-yEGFP-CYC1t) highlighted a remarkable drop in fluorescence both in the induced (41.20%) and uninduced (94.73%) conditions (Figure 4D). Overall, transcriptional interference proved to be a highly efficient mechanism for reducing protein synthesis.

### RNA interference from constitutive convergent promoters

**FIGURE 5:**
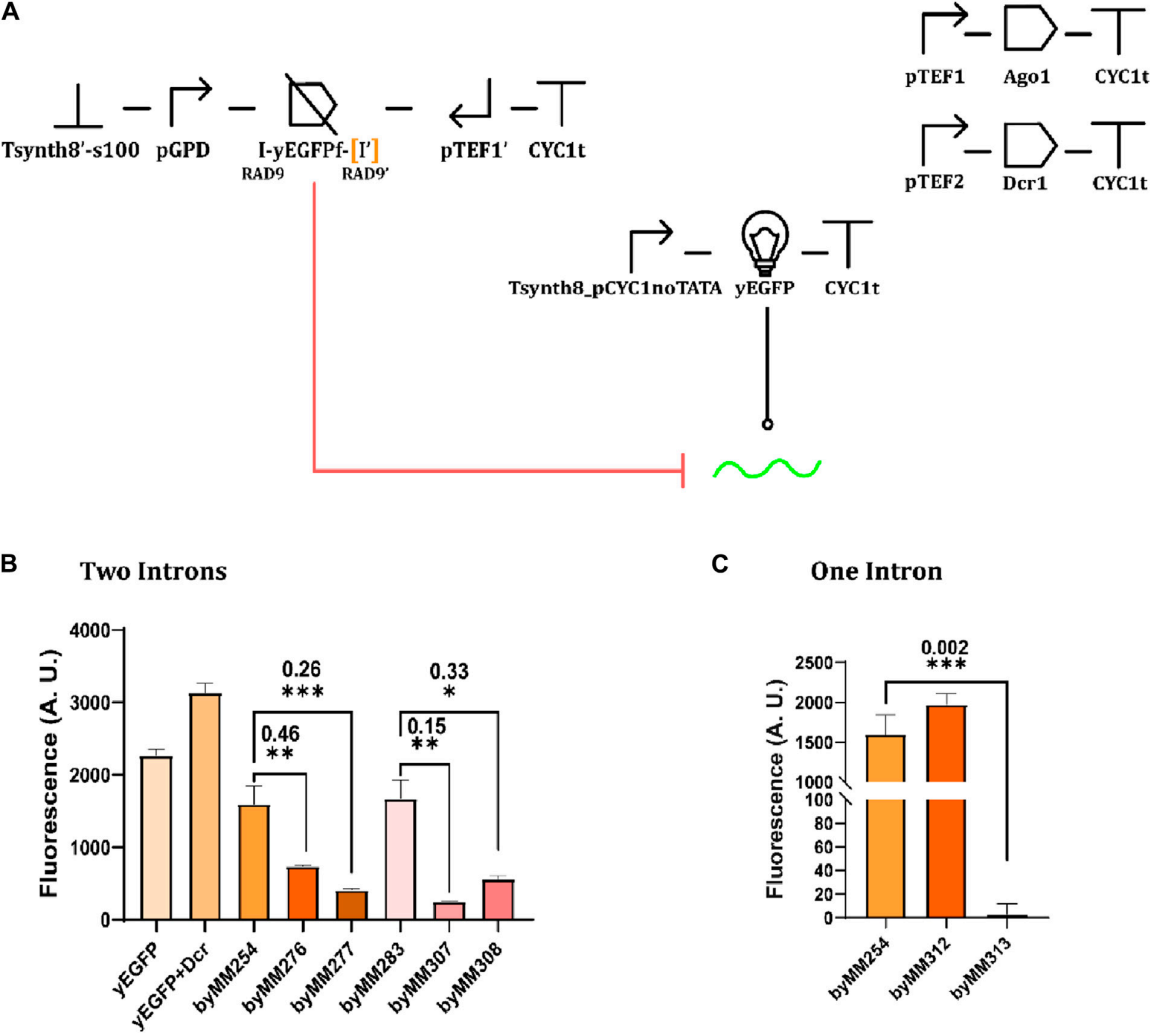
Convergent promoters expressing fragments of the yEGFP extended with one or two RAD9 introns. **(A)** Circuit diagram. yEGFPf indicates a fragment of the yEGFP. “I” represents an RAD9 intron. [I’] is the intron missing in the one-intron circuit. **(B)** Circuit performance in the presence of two RAD9 introns. Four complete circuits were constructed on two control strain (byMM254 and byMM283, sharing the same genetic content). Of these, byMM307 constructed on byMM283 demonstrated the most favorable outcome (Supplementary Table S11). **(C)** Circuit performance in the presence of a single RAD9 intron (*: *p*-value <0.05; **: *p*-value <0.01; ***: *p*-value< 0.001; two-sided Welch’s t-test).

After verifying the effects of transcriptional interference on protein expression, we tested the convergent promoter architecture inside circuits harnessing RNA interference. We used the TU in Figure 4A to express a fragment (Si et al., 2015; Crook et al., 2016) (200 nt) of the yEGFP (instead of the whole protein) along with one or two RAD9 introns (Drinnenberg et al., 2009; Crook et al., 2014), as depicted in Figure 5A. Introns are mainly adopted to improve RNA stability (Lewin et al., 2011). Convergent promoters lead to the synthesis of double-stranded sequences that resemble the siRNA precursor and are processed in siRNA molecules by Dcr1. We compared the fluorescence expressed by the complete circuit with that returned by the control strain (i.e., without convergent promoters). As shown in Figures 5B and C (and Supplementary Table S11), the two-intron yEGFP fragment design was effective in at least four strains, the best showing an OFF/ON ratio equal to 0.15. With the single-intron solution, in contrast, we achieved a remarkably low relative fluorescence (OFF/ON = 0.002), although in only one working strain (out of the two that grew on a selective medium after the last integration). Thus, the two-intron option appears more reliable, whereas the single-intron option is more potent but also harder to accomplish.

### RNAi-interference from galactose-inducible convergent promoters

**FIGURE 6:**
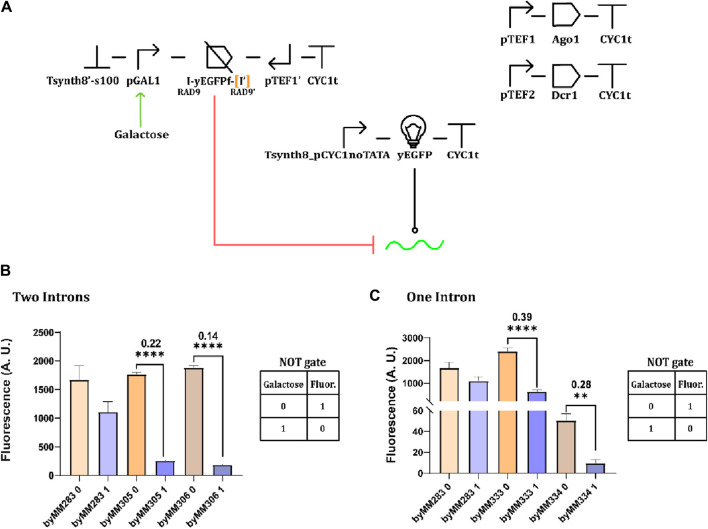
NOT gates based on galactose-inducible convergent promoters. Four complete circuits were constructed on the control strain byMM283. **(A)** Schematic NOT gate representation. **(B)** Circuit performance when two RAD9 introns flank the yEGFP fragment. Two circuits were obtained from the control strain byMM283: byMM305, which gave an OFF/ON ratio equal to 0.22, and byMM306 that reached OFF/ON = 0.14. **(C)** The best OFF/ON ratio in the presence of a single RAD9 intron corresponded to 0.28 (byMM334). However, the NOT gate in byMM333 (OFF/ON = 0.39) appeared to be less affected by pGAL1 leakage (**, *p*-value <0.01, ****: *p*-value< 0.0001; two-sided Welch’s t-test, see Supplementary Table S12).

After demonstrating that convergent promoters could be used to effectively silence protein synthesis, we replaced the galactose-inducible giant-hairpin expression cassette in the original circuit in Figure 1A with a galactose-inducible convergent-promoter system (where pGPD was replaced by pGAL1—see Figure 6A). The original circuit failed to work as a NOT gate because of pGAL1 leakage that provoked a dramatic drop in fluorescence even in the absence of galactose. In contrast, the convergent promoter architecture appeared much less affected by pGAL1 leakage. Efficient NOT gates were built using both one (OFF/ON ratio: 0.28 and 0.39) and two (0.14 and 0.22) RAD9 introns (Figures 6B, C).

### Boolean gates hosting constitutive convergent promoters

**FIGURE 7:**
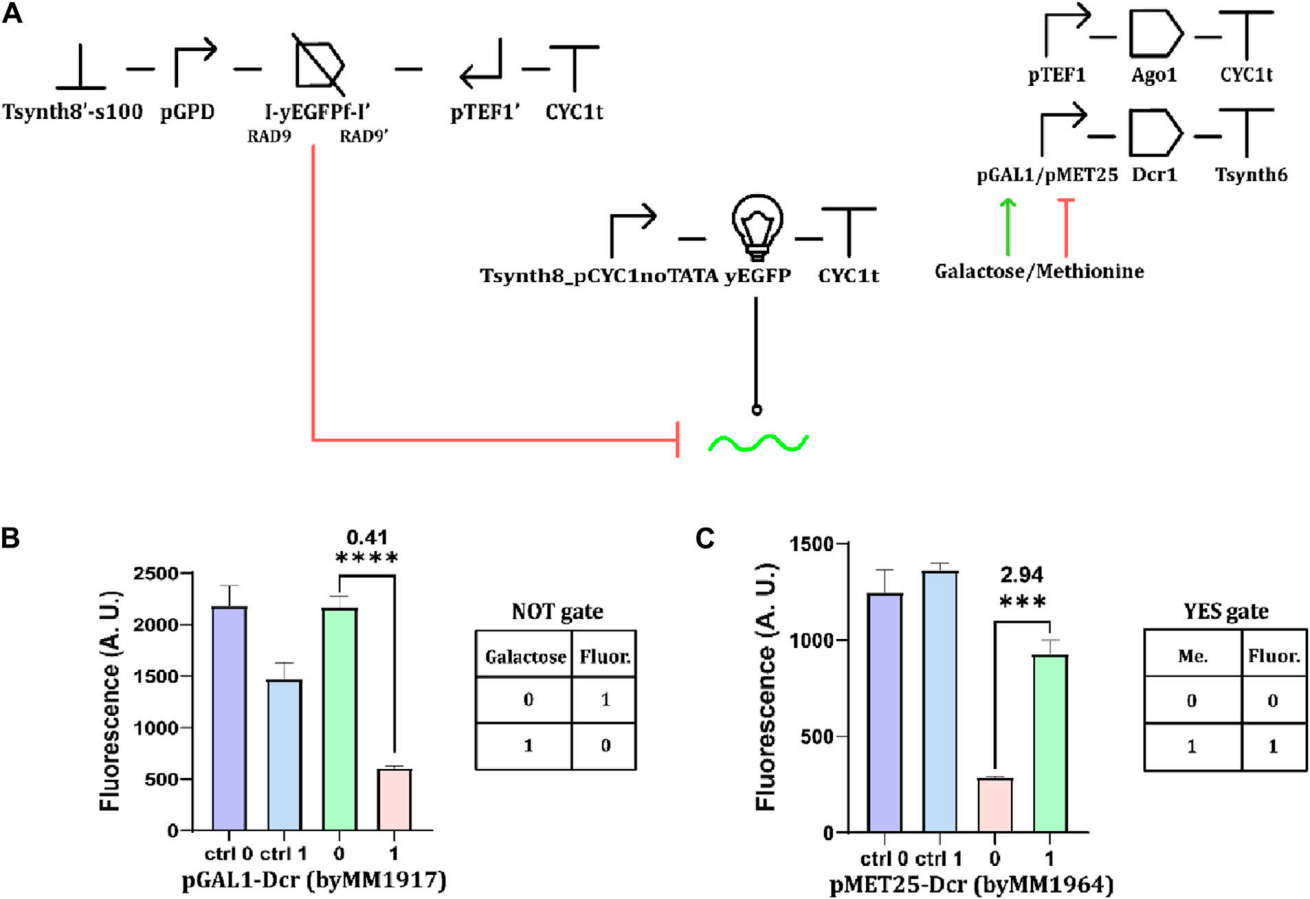
Basic Boolean gates hosting convergent promoters. **(A)** Schematic diagram of the circuits, incorporating convergent promoters and chemically regulated Dcr1 expression. Fluorescence intensity from **(B)** a NOT gate responding to 2% galactose and **(C)** a methionine-controlled YES gate (“1” level of methionine corresponded to a concentration of 10 mM ***: *p*-value< 0.001; ****: *p*-value <0.0001; two-sided Welch’s t-test, see Supplementary Table S13).

When dealing with a giant hairpin as a source of the siRNA precursor, we managed to implement working logic circuits only by expressing it constitutively and controlling the synthesis of the Dicer enzyme with an input chemical instead. We also applied this strategy to Boolean circuits hosting convergent promoters. In these tests, the yEGFP fragment was always accompanied by two RAD9 introns (Figure 7A). The new variant of the galactose-sensing NOT gate registered a moderately high OFF/ON ratio (0.41—see Figure 7B). The copper-responsive circuit failed to work as a Boolean gate because it returned the same fluorescence level in both the presence and absence of the CuSO_4_ (Supplementary Figure S7). In contrast, the methionine-sensing YES gate turned out to work effectively (ON/OFF ratio: 2.94—Figure 7C and Eq. 1 in Materials and Methods). Hence, promoter leakage seems to considerably affect the working of RNAi-based digital circuits even when they employ convergent promoters. The higher leakage of pCUP1 (compared to that of pMET25—see Supplementary Table S6) appears to be the most plausible reason for the failure in the construction of a NOT gate regulated by CuSO_4_ (Supplementary Figure S7).

**FIGURE 8:**
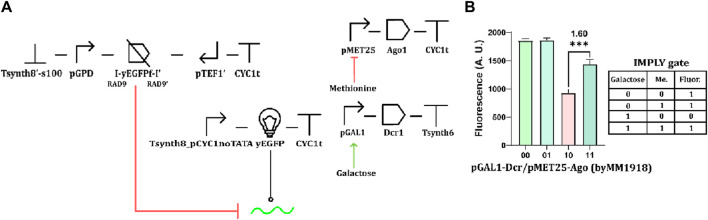
IMPLY gate based on convergent promoters. **(A)** Schematic representation of the circuit. **(B)** Fluorescence expression. 10 mM methionine and 2% galactose were used as “1” input values. (***: *p*-value <0.001; two-sided Welch’s t-test, see Supplementary Table S14).

Finally, we also implemented a version of the IMPLY gate that contained convergent promoters. Here, methionine repressed Ago1 production, whereas galactose induced the synthesis of Dcr1 expression (Figure 8A). The fluorescence levels returned by this implementation of the IMPLY gate effectively reflected the circuit truth table. However, the ρ value was rather low (1.60) because of the pMET25 leakage that determined a decrease in the fluorescence corresponding to the “11” truth-table entry (Figure 8B). This result further confirms the negative influence of promoter leakage in circuits whose function depends on the RNAi pathway.

### Generating an siRNA precursor via separate sense and antisense transcription of a DNA fragment

**FIGURE 9:**
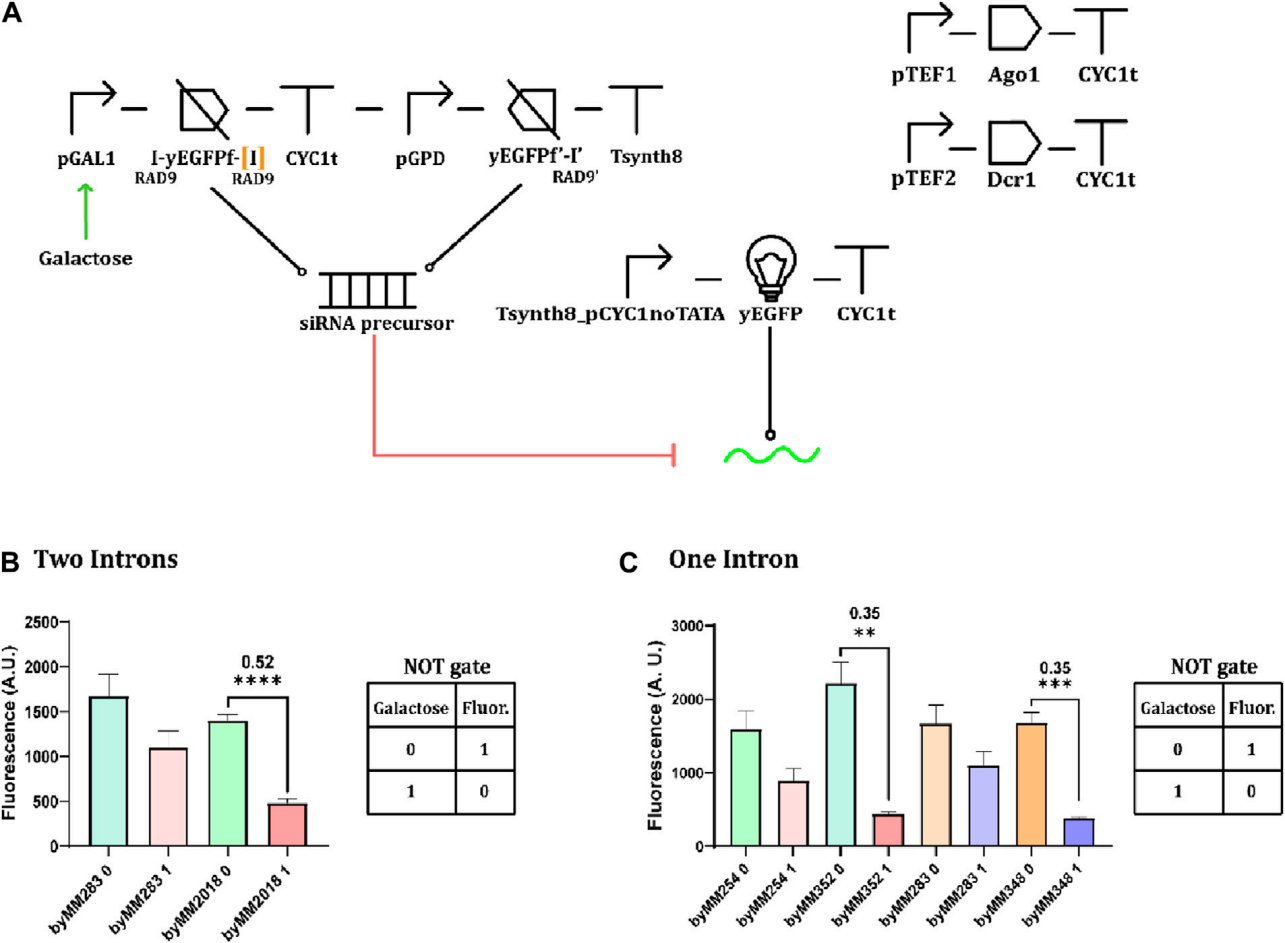
Galactose-responsive NOT gates with separate sense–antisense yEGFP fragments **(A)** NOT gate diagram. The sense fragment contains one or two introns. **(B)** Performance of a NOT gate hosting two RAD9 introns. The circuit was obtained from the control strain byMM283 and returned an OFF/ON ratio equal to 0.52. **(C)** Performance of NOT gates hosting a single RAD9 intron. Two circuits, byMM352 and byMM348, were obtained using byMM254 and byMM283 as control strains, respectively. They returned the same OFF/ON ratio: 0.35 (**, *p*-value <0.01, ***: *p*-value <0.001, ****: *p*-value <0.0001; two-sided Welch’s t-test, see Supplementary Table S15).

Another way to generate an siRNA precursor relies on two separate TUs that transcribe the same DNA fragment—one in the sense and the other in the antisense direction. This design, inspired by a similar one reported in Si et al. (2015), demands one more TU with respect to the circuits realized so far. As shown in Figure 9A, the sense DNA fragment (200 nt) of the yEGFP was produced upon induction with galactose, whereas the antisense fragment was constitutively synthesized by pGPD. Thus, in the presence of galactose, sense and antisense are transcribed at a very close rate. We designed two variants of this circuit that differ for the number of RAD9 introns (one or two) at the ends of the sense fragment. The antisense fragment always contains a single intron. Once transcribed, the sense and antisense transcripts pair and give rise to longer siRNA precursors than in the case of the convergent promoters. Overall, we engineered three strains hosting a complete NOT gate. As shown in Figure 9B, the two strains hosting a single intron upstream of the yEGFP sense fragment gave the best result, with an OFF/ON ratio equal to 0.35. Interestingly, this configuration was less effective than that based on the inducible convergent promoters shown in Figure 6, which is opposite to that obtained by Si et al. (2015), where the separate sense–antisense design was roughly five-fold stronger than the convergent promoters (all circuits were, however, uninduced). Si and colleagues did not, moreover, express a sense and antisense yEGFP fragment but the whole gene in both directions. Hence, their complete circuit comprised four TUs. In principle, we should have detected a fluorescence decrease compared to that of the control circuits (where the yEGFP-fragment-containing TUs were not present) already in the full circuit’s uninduced state. Indeed, in the absence of galactose, the antisense yEGFP fragment is transcribed and can bind by base-complementarity the full yEGFP mRNA, giving rise to a target for the Dicer enzyme. However, this did not occur. Therefore, we suppose that the efficiency of the separate sense–antisense design strongly depends on the length of the fragments—as it is also for the convergent promoters (Si et al., 2015).

## Conclusion

Back in 2009, Drinnenberg and colleagues (Drinnenberg et al., 2009) demonstrated how to reengineer the RNAi pathway in *S. cerevisiae*. They utilized a synthetic gene circuit in which siRNA molecules, which targeted the mRNA of a green fluorescent protein, should have only been produced in the presence of galactose—that is, upon activation of pGAL1 that led the transcription of the siRNA precursor. Even though RNAi was reestablished successfully, the study had a minor drawback since RNAi also occurred in the presence of glucose, when pGAL1 was almost completely repressed. In other words, the circuit failed to mimic a NOT gate that should have resulted in high fluorescence in the absence of galactose. Drinnenberg and colleagues suggested that either an antisense promoter or pGAL1 leakage could explain the issue. However, they did not investigate further as it was beyond the scope of their study. This marked the starting point of our study, which aimed to explore the use of RNAi in constructing basic Boolean gates in *S. cerevisiae* cells.

Through a series of tests, we confirmed that pGAL1 leakage, although minimal, completely disrupted the logic behavior of the original circuit by Drinnenberg and colleagues. By placing the Dicer enzyme under pGAL1 and expressing the giant hairpin siRNA precursor constitutively, we successfully constructed a working NOT and IMPLY gate. The former exhibited a remarkably low OFF/ON ratio, equal to 0.06. However, the number of strains transformed with a properly working logic gate hosting a giant hairpin-like siRNA precursor was consistently extremely low, if not zero. Hence, it was necessary to identify more reliable expression systems for the siRNA precursor that would not overly burden the engineered cells.

The convergent-promoter architecture guaranteed higher reliability and performance. In general, it appeared less susceptible to promoter leakage and performed well when the DNA fragment between the two promoters was expressed either constitutively or under chemical control. Additionally, gate performance could be modulated by including one or two introns in the siRNA-precursor-like sequence to enhance RNA stability. We also constructed NOT gates based on separate sense and antisense transcription of the same DNA fragment (see Table 1 for an overview of our best artifacts and results). This solution, however, required integrating one or more genes into the yeast genome than in the previous designs. Moreover, gate performance was lower than expected based on previously published data (Si et al., 2015). We hypothesize that the efficacy of this architecture depends heavily on the length of the DNA fragment, similar to observations with convergent promoters.

**TABLE 1:** Overview of top-performing gates constructed here.

Strains		Content	*p*-value	Ratio	Comments
Giant hairpin: inducible expression	byMM280	pGAL1-siRNA_precursor pTEF1-Ago1 pTEF2-Dcr1 Tsynth8_pCYC1noTATA-yEGFP	0.0001 (***)	0.62	- NOT gate - Too low ON state: 79.40 A.U. (control: 1597.60 A.U.)
	byMM1854	pGAL1-siRNA_precursor-poly(T) pTEF1-Ago1 pTEF2-Dcr1 Tsynth8_pCYC1noTATA-yEGFP	0.0177 (*)	0.29	- NOT gate - Too low ON state: 57.46 A.U. (control: 1441.40 A.U.)
Giant hairpin: inducible expression of the Dicer enzyme	byMM1699	pGPD-siRNA_precursor-Tsynth6 pTEF1-Ago1 pGAL1-Dcr1-Tsynth6 Tsynth8_pCYC1noTATA-yEGFP	0.0004 (***)	0.06	- NOT gate - ON state: 209.58 A.U. (control: 809.40 A.U.)
	byMM1864	pGPD-siRNA_precursor-Tsynth6 pTEF1-Ago1 pCUP1-Dcr1-Xbal-Tsynth6 Tsynth8_pCYC1noTATA-yEGFP	<0.0001 (****)	0.12	- NOT gate - Too low ON state: 36.45 A.U. (control: 1765.59 A.U.)
Giant hairpin: inducible expression of the Dcr and Ago	byMM1900	pGPD-siRNA_precursor-Tsynth6 pMET25-Ago1 pGAL1-Dcr1-Tsynth6 Tsynth8_pCYC1noTATA-yEGFP	0.0012 (**)	3.27 ( ρ -value)	- IMPLY gate - Low ON_L_ state: 142.50 A.U. (ON_H_ state: 981.51 A.U.)
Convergent promoter-based circuits	byMM307	Tsynth8’_sp100-pGPD-intronRAD9-yEGFPfragment-intronRAD9′-pTEF1’ pTEF1-Ago1 pTEF2- Dcr1 Tsynth8_pCYC1noTATA-yEGFP	0.0096 (**)	0.15	- No logic function
	byMM313	Tsynth8’_sp100-pGPD-intronRAD9-yEGFPfragment-pTEF1′ pTEF1-Ago1 pTEF2- Dcr1 Tsynth8_pCYC1noTATA-yEGFP	0.0001 (***)	0.002	- No logic function
	byMM306	Tsynth8’_sp100-pGAL1-intronRAD9-yEGFPfragment-intronRAD9′-pTEF1’ pTEF1-Ago1 pTEF2- Dcr1 Tsynth8_pCYC1noTATA-yEGFP	<0.0001 (****)	0.14	- NOT gate
	byMM333	Tsynth8’_sp100-pGAL1-intronRAD9-yEGFPfragment-pTEF1’ pTEF1-Ago1 pTEF2- Dcr1 Tsynth8_pCYC1noTATA-yEGFP	<0.0001 (****)	0.39	- NOT gate - High ON state: 2408.61 A.U. (control: 1673.64 A.U.)
	byMM1917	Tsynth8′-sp100-pGPD-intronRAD9-yEGFPfragment-intronRAD9′-pTEF1′ pTEF1-Ago1 pGAL1-Dcr1-Tsynth6 Tsynth8_pCYC1noTATA-yEGFP	<0.0001 (****)	0.41	- NOT gate
	byMM1964	Tsynth8′-sp100-pGPD-intronRAD9-yEGFPfragment-intronRAD9′-pTEF1′ pTEF1-Ago1 pMET25-Dcr1-Tsynth6 Tsynth8_pCYC1noTATA-yEGFP	0.0004 (***)	2.94	- YES gate
	byMM1918	Tsynth8′-sp100-pGPD-intronRAD9-yEGFPfragment-intronRAD9′-pTEF1’ pMET25-Ago1 pGAL1-Dcr1-Tsynth6 Tsynth8_pCYC1noTATA-yEGFP	0.0001 (***)	1.60 ( ρ -value)	- IMPLY gate Low ON_L_ state: 1437.37 A.U. (ON_H_ state: 1863.41 A.U.)
Sense and antisense transcription-based circuits	byMM2018	pGAL1-intronRAD9-yEGFPfragment-intronRAD9-CYC1t-pGPD-(RAD9-yEGFPfragment)’-Tsynth8 pTEF1-Ago1 pTEF2- Dcr1 Tsynth8_pCYC1noTATA-yEGFP	<0.0001 (****)	0.52	- NOT gate
	byMM348	pGAL1-intronRAD9-yEGFPfragment-CYC1t-pGPD-(intronRAD9-yEGFPfragment)’-Tsynth8 pTEF1-Ago1 pTEF2- Dcr1 Tsynth8_pCYC1noTATA-yEGFP	0.0003 (***)	0.35	- NOT gate

Overall, RNAi-based logic gates appear more susceptible to promoter leakage than transcriptional networks. Furthermore, they require more integration steps since the Argonaute and Dicer genes are absent from *S. cerevisiae* chromosomes. Even though they can guarantee high performance, particularly when introns are employed, their complex implementation has so far limited their widespread use in budding yeast synthetic biology.

## Materials and methods

### Plasmid construction

The plasmids utilized in this study are documented in Supplementary Table S16. These were constructed by employing the pRSII40X yeast integrating shuttle vector collection (Chee and Haase, 2012), where X denotes an auxotrophic marker. Specifically, we used four markers: HIS3, TRP1, LEU2, and URA3, corresponding to the following plasmids: pRSII403/Addgene-35436, pRSII404/Addgene-35438, pRSII405/Addgene-35440, and pRSII406/Addgene-35442, respectively (a gift from Steven Haase).

Two distinct methods were adopted in the construction of novel plasmids. The first involved enzymatic digestion and ligation. Initially, the backbone and the insert-containing plasmid were subjected to overnight digestion. Subsequently, the purified DNA fragments (extracted from the agarose gel with the AxyPrep DNA Gel Extraction Kit #AP-GX-250) were ligated by using T4 DNA ligase (NEB-M0202S) at 16 °C for more than 8 h. The second method for plasmid construction was isothermal assembly. Initially, standard biological parts including promoters, coding regions, and terminators were amplified via touchdown PCR using Q5 High-Fidelity DNA Polymerase (NEB-M0491S). The purified PCR products were then combined with a cut-open backbone (specifically, a pRSII40X plasmid devoid of the multiple cloning sequence) in equimolar quantities. This mixture was incubated in a thermal cycler at 50 °C for 1 h (Gibson et al., 2009).

All constructed plasmids in this study were introduced via a 30 s heat shock at 42 °C, into *Escherichia coli* cells (DH5α, Life Technology 18,263-012) and preserved in a glycerol storage solution. The DNA sequences of all DNA parts can be found in Supplementary Table S17. To ensure accuracy, each plasmid underwent Sanger sequencing at Genewiz Inc. in Suzhou, China.

### Yeast transformation

All genetically modified strains utilized in this research were derived from the *S. cerevisiae* strain CEN. PK2-1C (MATa; his3Δ1; leu2-3_112; ura3-52; trp1-289; MAL2-8c; SUC2) obtained from EUROSCARF (Johann Wolfgang Goethe University, Frankfurt, Germany—#30000A). The PEG/LiAc method was employed for yeast transformation (Gietz et al., 2002). Specifically, approximately 5 μg of integrative plasmid was linearized using a suitable restriction enzyme targeting the corresponding auxotrophic marker. Transformants were subsequently cultivated on a selective synthetic defined medium (SD, 2% glucose, 2% agar) at 30 °C for 2–3 days. Properly transformed strains were preserved in 15% glycerol storage solutions. A comprehensive list of all yeast strains generated in this investigation can be found in Supplementary Table S18.

### Fluorescence measurement

In the absence of an inducer in the growth medium, *S. cerevisiae* cells were cultured at 30 °C in a synthetic defined complete medium (SDC) supplemented with 2% glucose for 16 h. Conversely, when (2%) galactose was used as an inducer, the incubation period was extended to 24 h. For SDC supplemented with methionine (10 mM) or copper sulfate (0.5 mM), the incubation period ranged from 22 h to 24 h. Prior to fluorescence-activated cell sorting (FACS) experiments, the yeast cells were diluted 1:20 in SDC. Fluorescence intensity measurements were performed using a BD FACSVerse flow cytometer equipped with a blue laser (488 nm) and an emission filter (527/32). The FACS machine settings were validated through a quality check (QC) procedure utilizing fluorescent beads (BD FACS quite CS&T Research beads-17495). Each yeast strain was analyzed in triplicate on different days, representing independent experiments. In each experiment, 30,000 events were collected for each sample at low flow rates, ensuring a threshold rate below 2000 events per second. The resulting raw data obtained from the BD FACSVerse instrument were analyzed using the flowcore R-Bioconductor software package (Gietz et al., 2002).

### RT-qPCR

RNA extraction and purification from yeast cells were performed via the YeaStar RNA kit (Zymo Research-R1002). The obtained cDNA, which served as the template for qPCR, was synthesized using the Hifair III 1^st^ Strand cDNA Synthesis Kit (YEASEN-1141ES60). The primers used for amplifying yEGFP and the reference ACT1 gene are listed in Supplementary Table S19. For qPCR, a total volume of 20 μL qPCR solution was prepared, consisting of 10 μL Hieff qPCR SYBR Green Master Mix (No Rox) (YEASEN-11201ES08), 0.4 μL of 10 μM forward and reverse primers, a variable amount of cDNA (ranging from 20 to 50 ng), and RNase-free water. The following program was set up on a Roche LightCycler96 machine: (1) hold stage: 5 min at 95 °C; (2) PCR stage: 10 s at 95 °C, followed by 30 s at 60 °C. The PCR stage was repeated for 45 cycles. The threshold cycle (CT) values for ACT1 and yEGFP were determined using qPCR. Each sample was analyzed in triplicate. The relative mRNA expression levels were calculated using the Pfaffl formula (Pfaffl, 2001).

### Logic circuit analysis

NOT gates are components that convert a low input signal into a high output signal and *vice versa*. YES gates are devices that produce an output identical to their input. The evaluation of YES or NOT gates is commonly performed by calculating ON/OFF or OFF/ON ratios.

A complete or closed logic circuit (*Lc*) displays a binary output where fluorescence can be either high (“1”) or low (“0”). A control circuit (*Cc*) lacks at least a component that, in our case, prevents RNAi. Therefore, the fluorescence level of the control circuit should always be high.

The background fluorescence originating from the strain CEN. PK2-1C (byMM584), which serves as a chassis for our circuits, was subtracted from the average fluorescence value of both closed and control circuits. This adjusted fluorescence value will be denoted as F* in the formulae below.

A YES gate is a logic circuit that exhibits a high fluorescence value (ON state) when induced with an input chemical (*inp*) while displaying a low fluorescence value (OFF state) in the absence of the input from the growth medium (*grm*). To quantify the performance of a YES gate, the relative circuit fluorescence—the ON/OFF ratio—is calculated as follows (Yu and Marchisio, 2021):

$$\frac{ON}{OFF}=\frac{F_{Lc}^{*}\left(inp\right)}{F_{Cc}^{*}\left(inp\right)}\cdot \frac{F_{Cc}^{*}\left(grm\right)}{F_{Lc}^{*}\left(grm\right)}=\frac{F_{Lc}^{*}\left(inp\right)}{F_{Lc}^{*}\left(grm\right)}\cdot \frac{F_{Cc}^{*}\left(grm\right).}{F_{Cc}^{*}\left(inp\right)}$$ (1)

The bigger the ON/OFF ratio, the higher the efficiency of the circuit

A NOT gate is a logic circuit that returns a high fluorescence value (ON) in the absence of the input from the growth medium and a low fluorescence level (OFF) in the presence of the input. The performance of a NOT gate demands the calculation of the OFF/ON ratio:

$$\frac{OFF}{ON}=\frac{F_{Lc}^{*}\left(inp\right)}{F_{Lc}^{*}\left(grm\right)}\cdot \frac{F_{Cc}^{*}\left(grm\right)}{F_{Cc}^{*}\left(inp\right)}.$$ (2)

The lower the OFF/ON ratio, the higher the efficiency of the gate

Boolean gates that take *n* inputs (n > 1) are characterized by truth tables that are composed of 2^n^ entries, each associated with a high or low output (fluorescence level). The performance of the circuit is quantified with the ρ value (Marchisio, 2014) defined as:

$$\rho =\frac{F_{1L}^{*}}{F_{0H}^{*}}$$ (3)

where $F_{1L}^{*}$ represents the lowest “1” output and $F_{0H}^{*}$ the highest “0” output. A circuit is usually considered working if its ρ value is equal (or very close to) 2.

## Data Availability

FACS data are available at Flowrepository.org at the following links: http://flowrepository.org/id/RvFrJUacvKoYBpBliD5Mup1O7aZ9mG3fFgEIeQWjhFnU0ElLQhjdji3KrjNQQl7O http://flowrepository.org/id/RvFrrA8hlcPn1Xa036vZSC8yXYohfZxgFNbkVDqusly0blT9BKX9r4yOvvAdAii2

## References

[B1] Abraha B. W. Marchisio M. A. (2022). NOT gates based on protein degradation as a case study for a new modular modeling via SBML level 3—comp package. Front. Bioeng. Biotechnol. 10, 845240. 10.3389/fbioe.2022.845240 35360404 PMC8961978

[B2] Agrawal N. Dasaradhi P. Mohmmed A. Malhotra P. Bhatnagar R. K. Mukherjee S. K. (2003). RNA interference: biology, mechanism, and applications. Microbiol. Mol. Biol. Rev. 67, 657–685. 10.1128/mmbr.67.4.657-685.2003 14665679 PMC309050

[B3] Babiskin A. H. Smolke C. D. (2011). A synthetic library of RNA control modules for predictable tuning of gene expression in yeast. Mol. Syst. Biol. 7, 471. 10.1038/msb.2011.4 21364573 PMC3094065

[B4] Bellí G. Garí E. Piedrafita L. Aldea M. Herrero E. (1998). An activator/repressor dual system allows tight tetracycline-regulated gene expression in budding yeast [published erratum appears in Nucleic Acids Res 1998 Apr 1;26(7):following 1855]. Nucleic Acids Res. 26, 942–947. 10.1093/nar/26.4.942 9461451 PMC147371

[B5] Benenson Y. (2012). Biomolecular computing systems: principles, progress and potential. Nat. Rev. Genet. 13, 455–468. 10.1038/nrg3197 22688678

[B6] Blount B. A. Weenink T. Ellis T. (2012). Construction of synthetic regulatory networks in yeast. FEBS Lett. 586, 2112–2121. 10.1016/j.febslet.2012.01.053 22309848

[B7] Borchardt E. K. Vandoros L. A. Huang M. Lackey P. E. Marzluff W. F. Asokan A. (2015). Controlling mRNA stability and translation with the CRISPR endoribonuclease Csy4. RNA 21, 1921–1930. 10.1261/rna.051227.115 26354771 PMC4604432

[B8] Bordoy A. E. Varanasi U. S. Courtney C. M. Chatterjee A. (2016). Transcriptional interference in convergent promoters as a means for tunable gene expression. ACS Synth. Biol. 5, 1331–1341. 10.1021/acssynbio.5b00223 27346626

[B9] Brambilla A. Mainieri D. Carbone M. A. (1997). A simple signal element mediates transcription termination and mRNA 3′ end formation in the DEG1 gene of *Saccharomyces cerevisiae* . Mol. Gen. Genet. 254, 681–688. 10.1007/s004380050466 9202384

[B10] Chee M. K. Haase S. B. (2012). New and redesigned pRS plasmid shuttle vectors for genetic manipulation of *Saccharomyces cerevisiae* . G3 Genes., Genomes, Genet. 2, 515–526. 10.1534/g3.111.001917 PMC3362935 22670222

[B11] Cheng A. A. Lu T. K. (2012). Synthetic biology: an emerging engineering discipline. Annu. Rev. Biomed. Eng. 14, 155–178. 10.1146/annurev-bioeng-071811-150118 22577777

[B12] Crook N. Sun J. Morse N. Schmitz A. Alper H. S. (2016). Identification of gene knockdown targets conferring enhanced isobutanol and 1-butanol tolerance to *Saccharomyces cerevisiae* using a tunable RNAi screening approach. Appl. Microbiol. Biotechnol. 100, 10005–10018. 10.1007/s00253-016-7791-2 27654654

[B13] Crook N. C. Schmitz A. C. Alper H. S. (2014). Optimization of a yeast RNA interference system for controlling gene expression and enabling rapid metabolic engineering. ACS Synth. Biol. 3, 307–313. 10.1021/sb4001432 24328131

[B14] Curran K. A. Morse N. J. Markham K. A. Wagman A. M. Gupta A. Alper H. S. (2015). Short synthetic terminators for improved heterologous gene expression in yeast. ACS Synth. Biol. 4, 824–832. 10.1021/sb5003357 25686303

[B15] Drinnenberg I. A. Weinberg D. E. Xie K. T. Mower J. P. Wolfe K. H. Fink G. R. (2009). RNAi in budding yeast. Science 326, 544–550. 10.1126/science.1176945 19745116 PMC3786161

[B16] Farzadfard F. Perli S. D. Lu T. K. (2013). Tunable and multifunctional eukaryotic transcription factors based on CRISPR/Cas. ACS Synth. Biol. 2, 604–613. 10.1021/sb400081r 23977949 PMC3805333

[B17] Feldmann H. (2011) Yeast: molecular and cell biology. Hoboken: John Wiley & Sons.

[B18] Gibson D. G. Young L. Chuang R.-Y. Venter J. C. Hutchison III C. A. Smith H. O. (2009). Enzymatic assembly of DNA molecules up to several hundred kilobases. Nat. Methods 6, 343–345. 10.1038/nmeth.1318 19363495

[B19] Gietz R. D. Woods R. A. (2002). “Transformation of yeast by lithium acetate/single-stranded carrier DNA/polyethylene glycol method,” in Methods in enzymology. Editors Guthrie C. Fink G. R. (London: Academic Press), 87–96. 10.1016/s0076-6879(02)50957-5 12073338

[B20] Groher A.-C. Jager S. Schneider C. Groher F. Hamacher K. Suess B. (2018). Tuning the performance of synthetic riboswitches using machine learning. ACS Synth. Biol. 8, 34–44. 10.1021/acssynbio.8b00207 30513199

[B21] Han Z. Moore G. A. Mitter R. Martinez D. L. Wan L. Svejstrup A. B. D. (2023). DNA-directed termination of RNA polymerase II transcription. Mol. Cell. 83, 3253–3267.e7. 10.1016/j.molcel.2023.08.007 37683646 PMC7615648

[B22] Jackson J. S. Houshmandi S. S. Leban F. L. Olivas W. M. (2004). Recruitment of the Puf3 protein to its mRNA target for regulation of mRNA decay in yeast. RNA 10, 1625–1636. 10.1261/rna.7270204 15337848 PMC1370648

[B23] Kötter P. Weigand J. E. Meyer B. Entian K.-D. Suess B. (2009). A fast and efficient translational control system for conditional expression of yeast genes. Nucleic Acids Res. 37, e120. 10.1093/nar/gkp578 19592423 PMC2764425

[B24] Lam J. K. Chow M. Y. Zhang Y. Leung S. W. (2015). siRNA versus miRNA as therapeutics for gene silencing. Mol. Ther. Nucleic Acids 4, e252. 10.1038/mtna.2015.23 26372022 PMC4877448

[B25] Lewin B. Krebs J. Kilpatrick S. T. Goldstein E. S. (2011) Lewin's genes X. Burlington: Jones & Bartlett Learning.

[B26] Li J. Xu Z. Chupalov A. Marchisio M. A. (2018). Anti-CRISPR-based biosensors in the yeast *S. cerevisiae* . J. Biol. Eng. 12, 11–14. 10.1186/s13036-018-0101-z 30123320 PMC6090965

[B27] Liu Y. Ge H. Marchisio M. A. (2023). Hybrid Boolean gates show that Cas12c controls transcription activation effectively in the yeast *S. cerevisiae* . Front. Bioeng. Biotechnol. 11, 1267174. 10.3389/fbioe.2023.1267174 37771576 PMC10523329

[B28] Marchisio M. A. (2014). *In silico* design and in vivoimplementation of yeast gene Boolean gates. J. Biol. Eng. 8, 6–15. 10.1186/1754-1611-8-6 24485181 PMC3926364

[B29] Marchisio M. A. Stelling J. (2011). Automatic design of digital synthetic gene circuits. PLoS Comput. Biol. 7, e1001083. 10.1371/journal.pcbi.1001083 21399700 PMC3048778

[B30] Mazumder M. McMillen D. R. (2014). Design and characterization of a dual-mode promoter with activation and repression capability for tuning gene expression in yeast. Nucleic Acids Res. 42, 9514–9522. 10.1093/nar/gku651 25056312 PMC4132757

[B31] Møller T. S. Hay J. Saxton M. J. Bunting K. Petersen E. I. Kjærulff S. (2017). Human β-defensin-2 production from *S. cerevisiae* using the repressible MET17 promoter. Microb. Cell. Fact. 16, 11. 10.1186/s12934-017-0627-7 28100236 PMC5241953

[B32] Murphy K. F. Balázsi G. Collins J. J. (2007). Combinatorial promoter design for engineering noisy gene expression. Proc. Natl. Acad. Sci. 104, 12726–12731. 10.1073/pnas.0608451104 17652177 PMC1931564

[B33] Pfaffl M. W. (2001). A new mathematical model for relative quantification in real-time RT–PCR. Nucleic Acids Res. 29, e45. 10.1093/nar/29.9.e45 11328886 PMC55695

[B34] Rantasalo A. Kuivanen J. Penttila M. Jantti J. Mojzita D. (2018). Synthetic toolkit for complex genetic circuit engineering in *Saccharomyces cerevisiae* . ACS Synth. Biol. 7, 1573–1587. 10.1021/acssynbio.8b00076 29750501 PMC6150731

[B35] Regot S. Macia J. Conde N. Furukawa K. Kjellén J. Peeters T. (2011). Distributed biological computation with multicellular engineered networks. Nature 469, 207–211. 10.1038/nature09679 21150900

[B36] Semizarov D. Frost L. Sarthy A. Kroeger P. Halbert D. N. Fesik S. W. (2003). Specificity of short interfering RNA determined through gene expression signatures. Proc. Natl. Acad. Sci. 100, 6347–6352. 10.1073/pnas.1131959100 12746500 PMC164449

[B37] Shearwin K. E. Callen B. P. Egan J. B. (2005). Transcriptional interference–a crash course. Trends Genet. 21, 339–345. 10.1016/j.tig.2005.04.009 15922833 PMC2941638

[B38] Sheff M. A. Thorn K. S. (2004). Optimized cassettes for fluorescent protein tagging in *Saccharomyces cerevisiae* . Yeast 21, 661–670. 10.1002/yea.1130 15197731

[B39] Shetty R. S. Deo S. K. Liu Y. Daunert S. (2004). Fluorescence‐based sensing system for copper using genetically engineered living yeast cells. Biotechnol. Bioeng. 88, 664–670. 10.1002/bit.20331 15515160

[B40] Si T. Luo Y. Bao Z. Zhao H. (2015). RNAi-assisted genome evolution in *Saccharomyces cerevisiae* for complex phenotype engineering. ACS Synth. Biol. 4, 283–291. 10.1021/sb500074a 24758359

[B41] Sikorski R. S. Hieter P. (1989). A system of shuttle vectors and yeast host strains designed for efficient manipulation of DNA in *Saccharomyces cerevisiae* . Genetics 122, 19–27. 10.1093/genetics/122.1.19 2659436 PMC1203683

[B42] Song W. Li J. Liang Q. Marchisio M. A. (2016). Can terminators be used as insulators into yeast synthetic gene circuits? J. Biol. Eng. 10, 19–13. 10.1186/s13036-016-0040-5 28018483 PMC5162094

[B43] Sontheimer E. J. (2005). Assembly and function of RNA silencing complexes. Nat. Rev. Mol. Cell. Biol. 6, 127–138. 10.1038/nrm1568 15654322

[B44] Uwimana N. Collin P. Jeronimo C. Haibe-Kains B. Robert F. (2017). Bidirectional terminators in *Saccharomyces cerevisiae* prevent cryptic transcription from invading neighboring genes. Nucleic Acids Res. 45, 6417–6426. 10.1093/nar/gkx242 28383698 PMC5499651

[B45] Wang X.-H. Aliyari R. Li W.-X. Li H.-W. Kim K. Carthew R. (2006). RNA interference directs innate immunity against viruses in adult Drosophila. Science 312, 452–454. 10.1126/science.1125694 16556799 PMC1509097

[B46] Win M. N. Smolke C. D. (2008). Higher-order cellular information processing with synthetic RNA devices. Science 322, 456–460. 10.1126/science.1160311 18927397 PMC2805114

[B47] Yu L. Marchisio M. A. (2021). *Saccharomyces cerevisiae* synthetic transcriptional networks harnessing dCas12a and type VA anti-CRISPR proteins. ACS Synth. Biol. 10, 870–883. 10.1021/acssynbio.1c00006 33819020

[B48] Yu L. Marchisio M. A. (2023). CRISPR-associated type V proteins as a tool for controlling mRNA stability in *S. cerevisiae* synthetic gene circuits. Nucleic Acids Res. 51, 1473–1487. 10.1093/nar/gkac1270 36651298 PMC9943656

[B49] Zamore P. D. Tuschl T. Sharp P. A. Bartel D. P. (2000). RNAi: double-stranded RNA directs the ATP-dependent cleavage of mRNA at 21 to 23 nucleotide intervals. Cell. 101, 25–33. 10.1016/S0092-8674(00)80620-0 10778853

[B50] Zhang Y. Ge H. Marchisio M. A. (2022). A mutated Nme1Cas9 is a functional alternative RNase to both LwaCas13a and RfxCas13d in the yeast *S. cerevisiae* . Front. Bioeng. Biotechnol. 10, 922949. 10.3389/fbioe.2022.922949 35721864 PMC9201564

