# Supplementary material for: RNAi-based Boolean gates in the yeast Saccharomyces cerevisiae
Source: Front Bioeng Biotechnol. 2024 Jun 4;12:1392967. doi: 10.3389/fbioe.2024.1392967 (PMC11184144; doi:10.3389/fbioe.2024.1392967)
Supplement: Supplementary file 1 [file DataSheet1.PDF]

SUPPLEMENTARY MATERIAL

RNAi-based Boolean gates in the yeast *Saccharomyces cerevisiae*

Ximing Tian[1,+], Andrey Volkovinskiy[2,+], and Mario Andrea Marchisio[1,*]

[1] School of Pharmaceutical Science and Technology, Tianjin University, 92 Weijin Road, 300072 Tianjin, China

[2] School of Life Science and Technology, Harbin Institute of Technology, 2 Yikuang Street, 150080 Harbin, China

[+] the authors contributed equally

*corresponding author. Email address: mario@tju.edu.cn or mamarchisio@yahoo.com

Supplementary Figures

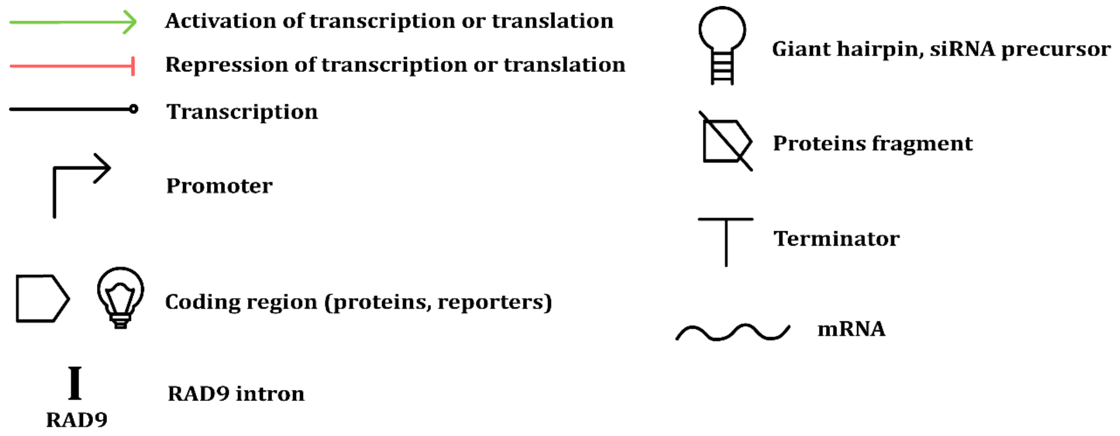

Figure S1. All symbols used in this work.

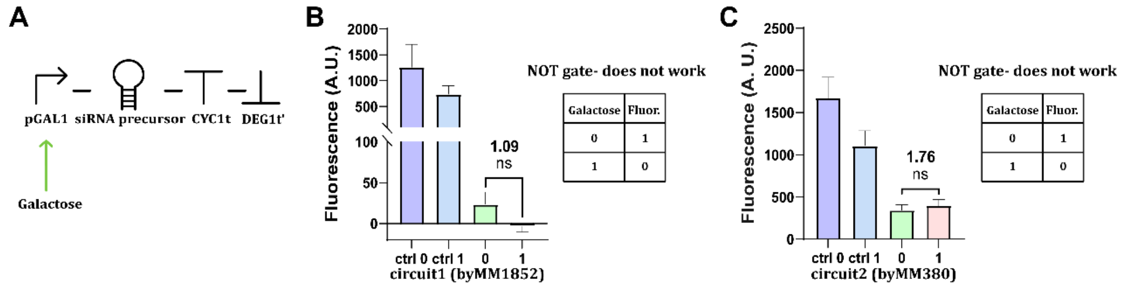

Figure S2. Inserting a DEG1 terminator on the antisense strand downstream of CYC1t. **(A)** Scheme of the modified transcription unit (TU) expressing the siRNA precursor as a giant hairpin. DEG1t' represents the reverse complement of DEG1t that should prevent RNA transcription from a downstream antisense promoter. **(B, C)** Fluorescence intensity from the two strains (byMM380 and byMM1852) hosting the same modified circuit. The presence of negative values is due to the subtraction, from the measured values, of the background noise, i.e., the fluorescence expressed by byMM584 (ns: no significant difference; two-sided Welch's t-test, see Table S3).

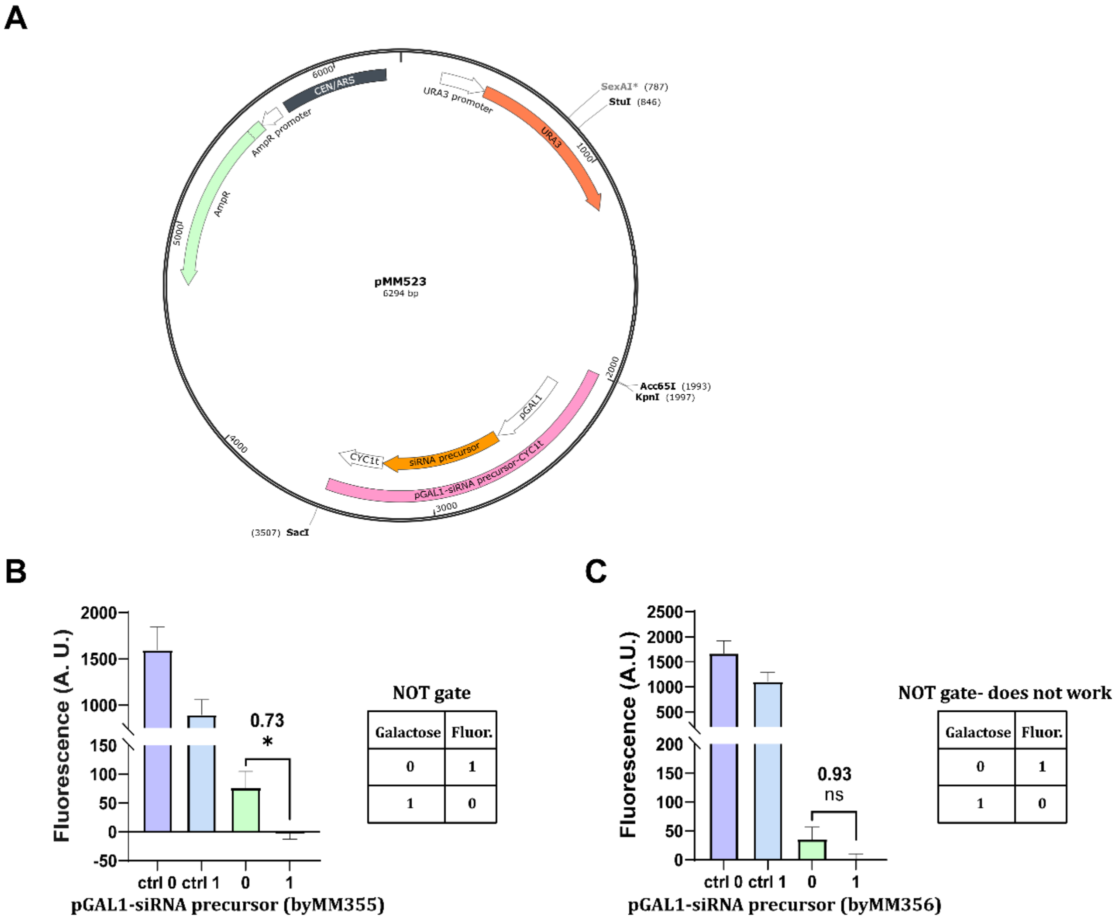

Figure S3. Circuit containing a centromeric plasmid. **(A)** Map of the the centromeric plasmid carrying the TU for the expression of the siRNA precursor. (**B, C**) Fluorescence intensity from the two strains (byMM355 and byMM356) harboring the complete circuit. Fluorescence intensity from the two strains (byMM380 and byMM1852) hosting the same modified circuit. The presence of negative values is due to the subtraction, from the measured values, of the background noise, i.e., the fluorescence expressed by byMM584. The NOT gate in byMM355 showed statistically significant different between the 0 and 1 output. However, the OFF/ON was too high, i.e., above the working threshold (0.5). (*: p-value < 0.05, ns: no significant difference; two-sided Welch's t-test, see Table S4).

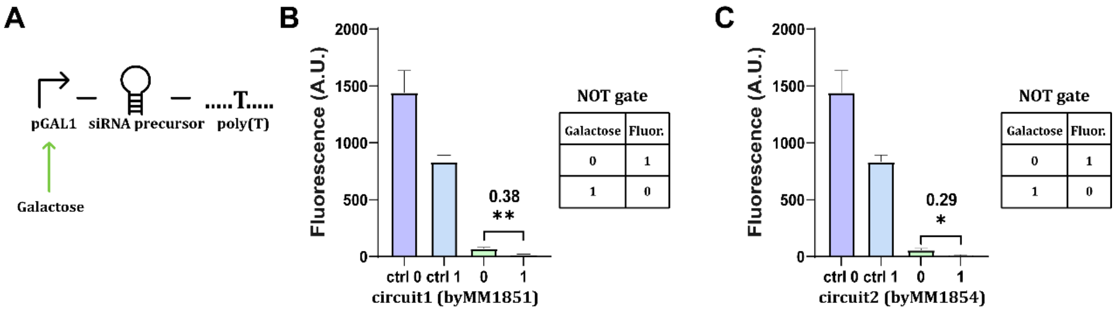

Figure S4. Replacing CYC1t with a poly(T) sequence. **(A)** Schematic representation of the modified TU for the synthesis of the siRNA precursor. **(B, C)** Fluorescence intensity. Both byMM1853 and byMM1854 strains harbor the complete circuit. In terms of statical difference and OFF/ON ratio, the two strains manage to mimic a NOT gate. However, the "1" output appear too low, if compared to the signal returned by the control circuit in the absence of galactose (*: p-value < 0.05; **: p-value < 0.01; two-sided Welch's t-test, see Table S5).

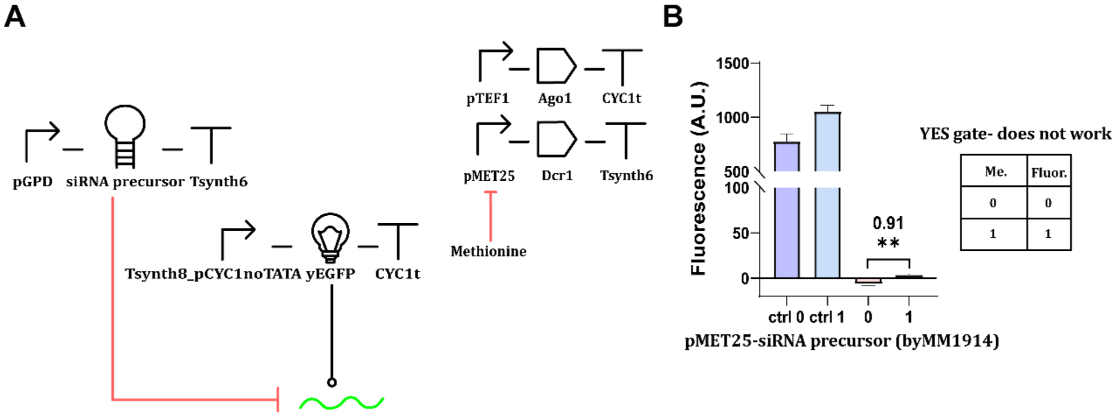

Figure S5. Repressible synthesis of Dcr1. **(A)** Schematic representation of a YES gates sensing methionine. pMET25 is placed in front of the *Dcr1* gene. **(B)** Circuit performance. The "1" concentration of methionine was set to 10 mM. The presence of negative values is due to the subtraction, from the measured values, of the background noise, i.e., the fluorescence expressed by byMM584 (**: p-value < 0.01; two-sided Welch's t-test, see Table S8).

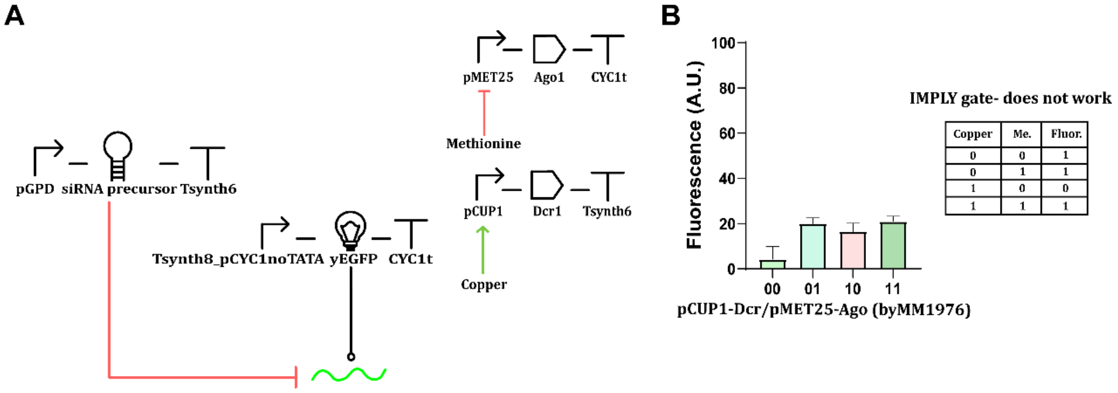

Figure S6. Tentative IMPLY gate design. **(A)** Circuit diagram. pCUP1 drives the synthesis of Dcr1 in the presence of $CuSO_4$, whereas pMET25 leads the production of Ago1 in the absence of methionine. **(B)** Fluorescence levels for different concentrations of $CuSO_4$ ("1" corresponds to 0.5 mM) and methionine ("1": 10 mM). The circuit implementation into the strain byMM1976 failed to reproduce a correct IMPLY logic function (*, p-value < 0.05; two-sided Welch' s t-test, see Table S9).

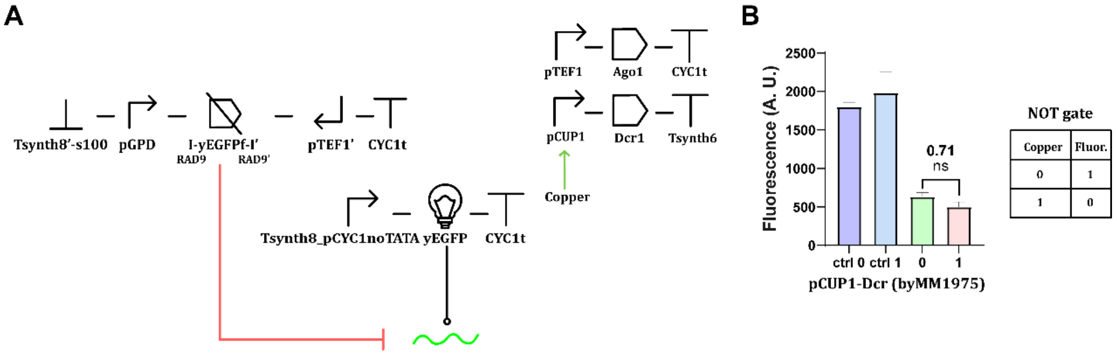

Figure S7. Hypothetical NOT gate sensing copper. **(A)** Circuit diagram. pCUP1 drives the synthesis of Dcr1 in the presence of $CuSO_4$. siRNAs come from the constitutive convergent promoters. The spacer100 is represented by the abbreviation s100, and the yEGFP fragment is denoted as yEGFPf. **(B)** Fluorescence levels for different concentrations of $CuSO_4$ ("1" corresponds to 0.5 mM) from strain byMM1975. The control circuit lacks the Dcr1 expression cassette (ns: no significant difference, p-value > 0.05; two-sided Welch' s t-test, see Table S13).

Supplementary Tables

Table S1. Mean green fluorescence intensity (FI) from both native and synthetic yeast promoters used in this work. Every value is the average from at least three independent measurements. SD: standard deviation. A.U.: arbitrary units.

**Promoter**	**Mean FI (A.U.)**	**SD (A.U.)**
pGAL1 (galactose)	16963.46	465.92
pGAL1 (glucose)	43.74	36.25
pTEF2	8063.26	56.68
pTEF1	2669.80	106.90
Tsynth8_pCYC1noTATA	2647.06	129.33
pGPD	18390.48	1210.51
pCUP1	672.00	115.58
pCUP1 (0.5 mM $CuSO_4$)	6420.62	568.39
pMET25	10158.02	348.82
pMET25 (10 mM met)	419.94	54.45

Table S2. Analysis of the circuit in Figure 1A. Mean fluorescence intensity (FI) and standard deviation (SD) are expressed in arbitrary units (A.U.) and obtained from a variable number of independent experiments (replicates) that were carried out in different days. The p-value was calculated using the two-sided Welch's t-test to compare mean FIs from the same (sub)circuit in the presence of galactose and glucose.

Strains	Content (every TU ends with CYC1t)		NOT GATE 0 1	NOT GATE 1 0	p-value	OFF/ON ratio
byMM584	—		—	—		
byMM234	Tsynth8_pCYC1noTATA-yEGFP	Mean(FI)	2268.04	—		
		SD	84.04			
		Replicates	3			
byMM250	pTEF2-Dcr1<br>Tsynth8_pCYC1noTATA-yEGFP	Mean(FI)	3133.35	—		
		SD	129.25			
		Replicates	3			
byMM254	pTEF1-Ago1<br>pTEF2-Dcr1<br>Tsynth8_pCYC1noTATA-yEGFP	Mean(FI)	1597.60	896.32	0.0011 (**)	0.58
		SD	244.52	162.69		
		Replicates	5	5		
byMM279	pGAL1-siRNA_precursor<br>pTEF1-Ago1<br>pTEF2-Dcr1<br>Tsynth8_pCYC1noTATA-yEGFP	Mean(FI)	27.55	-6.41	0.0001 (***)	1.08
		SD	9.95	4.30		
		Replicates	6	6		
byMM280	pGAL1-siRNA_precursor<br>pTEF1-Ago1<br>pTEF2-Dcr1<br>Tsynth8_pCYC1noTATA-yEGFP	Mean(FI)	79.40	-12.08	0.0006 (***)	0.62
		SD	22.66	5.76		
		Replicates	5	6		

Table S3. Analysis of the circuit in Figure S2. Mean fluorescence intensity (FI) and standard deviation (SD) are expressed in arbitrary units (A.U.) and obtained from a variable number of independent experiments (replicates) that were carried out in different days. The p-value was calculated using the two-sided Welch's t-test to compare mean FIs from the same (sub)circuit in the presence of galactose and glucose.

Strains	Content (every TU, unless otherwise specified, ends with CYC1t)		NOT GATE 0 1	1 0	p-value	OFF/ON ratio
byMM584	—		—	—		
byMM1849	pTEF1-Ago1<br>pTEF2-Dcr1<br>Tsynth8-pCYC1noTATA-yEGFP	Mean(FI)	1262.70	741.20	0.17 (ns)	0.60
		SD	439.51	162.11		
		Replicates	3	3		
byMM1852	pGAL1-siRNA_precursor-CYC1t-DEG1t'<br>pTEF1-Ago1<br>pTEF2-Dcr1<br>Tsynth8_pCYC1noTATA-yEGFP	Mean(FI)	23.64	-3.41	0.070 (ns)	1.09
		SD	14.94	7.06		
		Replicates	3	3		
byMM283	pTEF1-Ago1<br>pTEF2-Dcr1<br>Tsynth8_pCYC1noTATA-yEGFP	Mean(FI)	1673.64	1106.07	0.0022 (**)	0.66
		SD	245.01	179.44		
		Replicates	7	4		
byMM380	pGAL1-siRNA_precursor-CYC1t-DEG1t'<br>pTEF1-Ago1<br>pTEF2-Dcr1<br>Tsynth8_pCYC1noTATA-yEGFP	Mean(FI)	341.52	341.52	0.36 (ns)	1.76
		SD	64.77	70.19		
		Replicates	3	3		

Table S4. Analysis of the circuit in Figure S3. Mean fluorescence intensity (FI) and standard deviation (SD) are expressed in arbitrary units (A.U.) and obtained from a variable number of independent experiments (replicates) that were carried out in different days. The p-value was calculated using the two-sided Welch's t-test to compare mean FIs from the same (sub)circuit in the presence of galactose and glucose.

Strains	Content (every TU, unless otherwise specified, ends with CYC1t)		NOT GATE		p-value	OFF/ON ratio
			0<br>1	1<br>0		
byMM584	—		—	—		
byMM254	pTEF1-Ago1<br>pTEF2-Dcr1<br>Tsynth8_pCYC1noTATA-yEGFP	Mean(FI)	1597.60	896.32	0.0011<br>(**)	0.58
		SD	244.52	162.69		
		Replicates	5	5		
byMM355	pGAL1-siRNA_precursor<br>pTEF1-Ago1<br>pTEF2- Dcr1<br>Tsynth8_pCYC1noTATA-yEGFP	Mean(FI)	76.55	-3.83	0.029<br>(*)	0.71
		SD	28.17	9.22		
		Replicates	3	3		
byMM283	pTEF1-Ago1<br>pTEF2-Dcr1<br>Tsynth8_pCYC1noTATA-yEGFP	Mean(FI)	1673.64	1106.07	0.0022<br>(**)	0.66
		SD	245.01	179.44		
		Replicates	7	4		
byMM356	pGAL1-siRNA_precursor<br>pTEF1-Ago1<br>pTEF2- Dcr1<br>Tsynth8_pCYC1noTATA-yEGFP	Mean(FI)	35.71	0.8	0.079<br>(ns)	0.93
		SD	20.42	9.71		
		Replicates	3	3		

Table S5. Analysis of the circuit in Figure S4. Mean fluorescence intensity (FI) and standard deviation (SD) are expressed in arbitrary units (A.U.) and obtained from a variable number of independent experiments (replicates) that were carried out in different days. The p-value was calculated using the two-sided Welch's t-test to compare mean FIs from the same (sub)circuit in the presence of galactose and glucose.

Strains	Content (every TU, unless otherwise specified, ends with CYC1t)		NOT GATE 0<br>1	1<br>0	p-value	OFF/ON ratio
byMM584	—		—	—		
byMM1849	pTEF1-Ago1<br>pTEF2-Dcr1<br>Tsynth8_pCYC1noTATA-yEGFP	Mean(FI)	1441.40	832.31	0.024 (**)	0.58
		SD	192.35	59.20		
		Replicates	3	3		
byMM1851	pGAL1-siRNA_precursor-poly(T)<br>pTEF1-Ago1<br>pTEF2-Dcr1<br>Tsynth8_pCYC1noTATA-yEGFP	Mean(FI)	71.08	15.58	0.0024 (**)	0.38
		SD	9.02	5.28		
		Replicates	3	4		
byMM1854	pGAL1-siRNA_precursor-poly(T)<br>pTEF1-Ago1<br>pTEF2-Dcr1<br>Tsynth8_pCYC1noTATA-yEGFP	Mean(FI)	57.46	9.73	0.0177 (*)	0.29
		SD	13.49	6.11		
		Replicates	3	6		

Table S6. Promoter leakage determined from FACS and RT-qPCR experiments.

Promoter	Mean FI ± SD (A.U.)	State	Leakage (%)	mRNA relative level	Leakage (%)
pGAL1 (galactose)	16963.46 ± 465.92	ON		4.34	
pGAL1 (glucose)	43.74 ± 36.25	OFF	0.26	0.02	0.46
pCUP1 (0.5 mM CuSO4)	6420.62 ± 568.39	ON		-	
pCUP1	672.00 ± 115.58	OFF	10.47	-	-
pMET25	10158.02 ± 348.82	ON		1.96	
pMET25 (10 mM met)	419.94 ± 54.45	OFF	4.13	0.17	8.67

Table S7. Analysis of the NOT gates in Figure 2. Mean fluorescence intensity (FI) and standard deviation (SD) are expressed in arbitrary units (A.U.) and obtained from a variable number of independent experiments (replicates) that were carried out in different days. The p-value was calculated using the two-sided Welch's t-test to compare mean FIs from the same (sub)circuit in the presence/absence of galactose ($CuSO_4$).

Strains	Content (every TU, unless otherwise specified, ends with CYC1t)		NOT GATE		p-value	OFF/ON ratio
			0<br>1	1<br>0		
byMM584	—		—	—		
byMM1696	pTEF1-Ago1<br>pGAL1-Dcr1-Tsynth6<br>Tsynth8_pCYC1noTATA-yEGFP	Mean(FI)	809.40	704.44	0.42<br>(ns)	0.87
		SD	177.43	59.31		
		Replicates	3	3		
byMM1699	pGPD-siRNA_precursor-Tsynth6<br>pTEF1-Ago1<br>pGAL1-Dcr1-Tsynth6<br>Tsynth8_pCYC1noTATA-yEGFP	Mean(FI)	209.58	15.15	0.0004<br>(***)	0.06
		SD	13.61	3.83		
		Replicates	3	3		
byMM1846	pTEF1-Ago1<br>pCUP1-Dcr1-Tsynth6<br>Tsynth8_pCYC1noTATA-yEGFP	Mean(FI)	1765.59	3053.22	0.0035<br>(**)	1.73
		SD	70.30	340.16		
		Replicates	3	4		
byMM1864	pGPD-siRNA_precursor-Tsynth6<br>pTEF1-Ago1<br>pCUP1-Dcr1 -Tsynth6<br>Tsynth8_pCYC1noTATA-yEGFP	Mean(FI)	36.45	7.29	<0.0001<br>(****)	0.12
		SD	5.28	3.53		
		Replicates	5	5		

Table S8. Analysis of the YES gate in Figure S5. Mean fluorescence intensity (FI) and standard deviation (SD) are expressed in arbitrary units (A.U.) and obtained from a variable number of independent experiments (replicates) that were carried out in different days. The p-value was calculated using the two-sided Welch's t-test to compare mean FIs from the same (sub)circuit in the presence/absence of methionine. The gate working performance is evaluated via the ON/OFF ratio.

Strains	Content (every TU, unless otherwise specified, ends with CYC1t)		YES GATE		p-value	ON/OFF ratio
			1<br>1	0<br>0		
byMM584	—		—	—		
byMM1897	pTEF1-Ago1<br>pMET25-Dcr1-Tsynth6<br>Tsynth8_pCYC1noTATA-yEGFP	Mean(FI)	1054.36	778.36	0.0071 (**)	1.33
		SD	58.20	70.56		
		Replicates	3	3		
byMM1914	pGPD-siRNA_precursor-Tsynth6<br>pTEF1-Ago1<br>pMET25-Dcr1-Tsynth6<br>Tsynth8_pCYC1noTATA-yEGFP	Mean(FI)	4.02	-6.55	0.0062 (**)	0.91
		SD	0.67	1.94		
		Replicates	3	3		

Table S9 Analysis of the IMPLY gate in Figure 3 and the circuit in Figure S6. Mean fluorescence intensity (FI) and standard deviation (SD) are expressed in arbitrary units (A.U.) and obtained from a variable number of independent experiments (replicates) that were carried out in different days. The p-value was calculated using the two-sided Welch's t-test to compare mean FIs from the same (sub)circuit in the smallest output of "1" and the largest output of "0". Circuit performance is evaluated via the ρ value.

Strains	Content (every TU, unless otherwise specified, ends with CYC1t)		IMPLY GATE				p-value	ρ-value
			1<br>00	1<br>01	0<br>10	1<br>11		
byMM584	—		—	—	—	—		
byMM1900	pGPD-siRNA_precursor-Tsynth6<br>pMET25-Ago1<br>pGAL1-Dcr1-Tsynth6<br>Tsynth8_pCYC1noTATA-yEGFP	Mean(FI)	480.55	981.51	43.60	142.50	0.0012 (**)	3.27
		SD	31.07	55.50	14.50	14.99		
		Replicates	3	5	3	3		
byMM1976	pGPD-siRNA_precursor-Tsynth6<br>pMET25-Ago1<br>pCUP1-Dcr1-Tsynth6<br>Tsynth8_pCYC1noTATA-yEGFP	Mean(FI)	4.21	19.96	16.49	20.96	-	-
		SD	5.68	2.55	3.74	2.32		
		Replicates	3	3	3	3		

Table S10. Analysis of circuits based on convergent promoters illustrated in Figure 4. Mean fluorescence intensity (FI) and standard deviation (SD) are expressed in arbitrary units (A.U.) and obtained from a variable number of independent experiments (replicates) that were carried out in different days. The p-value was calculated using the two-sided Welch's t-test to compare mean FIs from the same circuit in the presence of galactose and glucose.

Strains	Content (every TU, unless otherwise specified, ends with CYC1t)	Mean(FI)	SD	Replicates
byMM584	—	—	—	—
byMM282	Tsynth8'_spacer100-pGPD-yEGFP-pTEF1'	9898.011602	769.2703817	5
byMM103	pGPD-yEGFP	18667.34531	569.5381642	3

Strains	Content (every TU, unless otherwise specified, ends with CYC1t)		YES GATE		p-value
			0	1	
			0	1	
byMM584	—		—	—	—
byMM224	pGAL1-yEGFP	Mean(FI)	55.97	16106.38	0.0004 (***)
		SD	4.24	524.83	
		Replicates	3	3	
byMM292	Tsynth8'_spacer100-pGAL1-yEGFP-pTEF1'	Mean(FI)	2.95	9470.16	<0.0001 (****)
		SD	1.81	282.32	
		Replicates	3	4	

Table S11. Analysis of circuits, based on convergent promoters, illustrated in Figure 5. Mean fluorescence intensity (FI) and standard deviation (SD) are expressed in arbitrary units (A.U.) and obtained from a variable number of independent experiments (replicates) that were carried out in different days. The p-value was calculated using the two-sided Welch's t-test to compare mean FIs from the full and the control circuit. Circuit performance is quantified with the complete/control circuit ratio.

Strains	Content<br>(every TU, unless otherwise specified, ends with CYC1t)	Mean(FI)	SD	Replicates	p-value	ratio
byMM584	—	—	—	—		
byMM254	pTEF1-Ago1<br>pTEF2-Dcr1<br>Tsynth8_pCYC1noTATA-yEGFP	1597.60	244.52	5	—	—
byMM276	Tsynth8'_sp100-pGPD-intronRAD9-yEGFPfragment-intronRAD9'-pTEF1'<br>pTEF1-Ago1<br>pTEF2- Dcr1<br>Tsynth8_pCYC1noTATA-yEGFP	736.16	20.63	3	0.0013<br>(**)	0.46
byMM277	Tsynth8'_sp100-pGPD-intronRAD9-yEGFPfragment-intronRAD9'-pTEF1'<br>pTEF1-Ago1<br>pTEF2- Dcr1<br>Tsynth8_pCYC1noTATA-yEGFP	409.91	15.21	3	0.0004<br>(***)	0.26
byMM283	pTEF1-Ago1<br>pTEF2-Dcr1<br>Tsynth8_pCYC1noTATA-yEGFP	1673.64	245.01	7	—	—
byMM307	Tsynth8'_sp100-pGPD-intronRAD9-yEGFPfragment-intronRAD9'-pTEF1'<br>pTEF1-Ago1<br>pTEF2- Dcr1<br>Tsynth8_pCYC1noTATA-yEGFP	249.85	11.00	3	0.0096<br>(**)	0.15
byMM308	Tsynth8'_sp100-pGPD-intronRAD9-yEGFPfragment-intronRAD9'-pTEF1'<br>pTEF1-Ago1<br>pTEF2- Dcr1<br>Tsynth8_pCYC1noTATA-yEGFP	557.62	45.06	4	0.0139<br>(*)	0.33

Strains	Content<br>(every TU, unless otherwise specified, ends with CYC1t)	Mean(FI)	SD	Replicates	p-value	ratio
byMM584	—	—	—	—		
byMM254	pTEF1-Ago1<br>pTEF2-Dcr1<br>Tsynth8_pCYC1noTATA-yEGFP	1597.60	244.52	5	—	—

byMM312	Tsynth8'_sp100-pGPD-intronRAD9-yEGFPfragment-pTEF1'<br>pTEF1-Ago1<br>pTEF2- Dcr1<br>Tsynth8_pCYC1noTATA-yEGFP	1969.80	134.03	3	0.0321 (*)	1.23
byMM313	Tsynth8'_sp100-pGPD-intronRAD9-yEGFPfragment-pTEF1'<br>pTEF1-Ago1<br>pTEF2- Dcr1<br>Tsynth8_pCYC1noTATA-yEGFP	3.26	8.47	5	0.0001 (***)	0.002

Table S12. Analysis of NOT gates based on inducible-convergent promoters illustrated in Figure 6. Mean fluorescence intensity (FI) and standard deviation (SD) are expressed in arbitrary units (A.U.) and obtained from a variable number of independent experiments (replicates) that were carried out in different days. The p-value was calculated using the two-sided Welch's t-test to compare mean FIs from the same (sub)circuit in the presence/absence of galactose. Gate performance is quantified with OFF/ON ratio.

Strains	Content (every TU, unless otherwise specified, ends with CYC1t)		NOT GATE 0 / 1	NOT GATE 1 / 0	p-value	OFF/ON ratio
byMM584	—		—	—		
byMM283	pTEF1-Ago1<br>pTEF2-Dcr1<br>Tsynth8_pCYC1noTATA-yEGFP	Mean(FI)	1673.64	1106.17	0.0022 (**)	0.66
		SD	245.01	179.45		
		Replicates	7	4		
byMM305	Tsynth8’_sp100-pGAL1-intronRAD9-yEGFPfragment-intronRAD9’-pTEF1'<br>pTEF1-Ago1<br>pTEF2- Dcr1<br>Tsynth8_pCYC1noTATA-yEGFP	Mean(FI)	1766.48	257.29	<0.0001 (****)	0.22
		SD	29.14	8.91		
		Replicates	3	3		
byMM306	Tsynth8’_sp100-pGAL1-intronRAD9-yEGFPfragment-intronRAD9’-pTEF1'<br>pTEF1-Ago1<br>pTEF2- Dcr1<br>Tsynth8_pCYC1noTATA-yEGFP	Mean(FI)	1882.01	180.96	<0.0001 (****)	0.14
		SD	38.93	14.37		
		Replicates	3	4		

Strains	Content (every TU, unless otherwise specified, ends with CYC1t)		NOT GATE 0 / 1	NOT GATE 1 / 0	p-value	OFF/ON ratio
byMM584	—		—	—		
byMM283	pTEF1-Ago1<br>pTEF2-Dcr1<br>Tsynth8_pCYC1noTATA-yEGFP	Mean(FI)	1673.64	1106.17	0.0022 (**)	0.66
		SD	245.01	179.44		
		Replicates	7	4		
byMM333	Tsynth8’_sp100-pGAL1-intronRAD9-yEGFPfragment-pTEF1'<br>pTEF1-Ago1<br>pTEF2- Dcr1<br>Tsynth8_pCYC1noTATA-yEGFP	Mean(FI)	2408.61	626.99	<0.0001 (****)	0.39
		SD	144.76	76.079		
		Replicates	4	3		
byMM334	Tsynth8’_sp100-pGAL1-intronRAD9-yEGFPfragment-pTEF1'<br>pTEF1-Ago1<br>pTEF2- Dcr1<br>Tsynth8_pCYC1noTATA-yEGFP	Mean(FI)	50.68	9.50	0.0017 (**)	0.28
		SD	6.20	3.53		
		Replicates	3	3		

Table S13. Analysis of the YES and NOT gates in Figure 7 and the circuit in Figure S7. Mean fluorescence intensity (FI) and standard deviation (SD) are expressed in arbitrary units (A.U.) and obtained from a variable number of independent experiments (replicates) that were carried out in different days. The p-value was calculated using the two-sided Welch's t-test to compare mean FIs from the circuit in the presence and absence of the input chemical. Gate performance is quantified with the ON/OFF (YES) or OFF/ON (NOT) ratio.

Strains	Content<br>(every TU, unless otherwise specified, ends with CYC1t)		NOT GATE		p-value	OFF/ON ratio
			0	1		
			1	0		
byMM584	—		—	—		
byMM1912	Tsynth8'-sp100-pGPD-intronRAD9-yEGFPfragment-intronRAD9'-pTEF1'<br>pTEF1-Ago1<br>Tsynth8_pCYC1noTATA-yEGFP	Mean(FI)	2180.70	1474.66	0.0033 (**)	0.68
		SD	196.64	155.86		
		Replicates	4	3		
byMM1917	Tsynth8'-sp100-pGPD-intronRAD9-yEGFPfragment-intronRAD9'-pTEF1'<br>pTEF1-Ago1<br>pGAL1-Dcr1-Tsynth6<br>Tsynth8_pCYC1noTATA-yEGFP	Mean(FI)	2169.36	608.74	<0.0001 (****)	0.41
		SD	2169.36	18.76		
		Replicates	5	3		
byMM1912	Tsynth8'-sp100-pGPD-intronRAD9-yEGFPfragment-intronRAD9'-pTEF1'<br>pTEF1-Ago1<br>Tsynth8_pCYC1noTATA-yEGFP	Mean(FI)	1798.44	1983.28	0.35 (ns)	1.10
		SD	54.26	267.15		
		Replicates	3	3		
byMM1975	Tsynth8'-sp100-pGPD-intronRAD9-yEGFPfragment-intronRAD9'-pTEF1'<br>pTEF1-Ago1<br>pCUP1-Dcr1-Tsynth6<br>Tsynth8_pCYC1noTATA-yEGFP	Mean(FI)	635.04	500.28	0.0533 (ns)	0.71
		SD	52.16	66.12		
		Replicates	3	3		

Strains	Content<br>(every TU, unless otherwise specified, ends with CYC1t)		YES GATE		p-value	ON/OFF ratio
			1	0		
			1	0		
byMM584	—		—	—		
byMM1912	Tsynth8'-sp100-pGPD-intronRAD9-yEGFPfragment-intronRAD9'-pTEF1'<br>pTEF1-Ago1<br>Tsynth8_pCYC1noTATA-yEGFP	Mean(FI)	1249.04	1364.86	0.1418 (ns)	1.09
		SD	117.70	33.83		
		Replicates	4	4		
byMM1964	Tsynth8'-sp100-pGPD-intronRAD9-yEGFPfragment-intronRAD9'-pTEF1'<br>pTEF1-Ago1<br>pMET25-Dcr1-Tsynth6<br>Tsynth8_pCYC1noTATA-yEGFP	Mean(FI)	288.35	927.32	0.0004 (***)	2.94
		SD	4.09	72.32		
		Replicates	3	4		

Table S14. Analysis of the IMPLY gate in Figure 8. Mean fluorescence intensity (FI) and standard deviation (SD) are expressed in arbitrary units (A.U.) and obtained from a variable number of independent experiments (replicates) that were carried out in different days. The p-value was calculated using the two-sided Welch's t-test to compare lowest "1" fluorescence level with the only "0" output. The gate performance is quantified by the ρ-value.

Strains	Content (every TU, unless otherwise specified, ends with CYC1t)		IMPLY GATE 1 00	1 01	0 10	1 11	p-value	ρ-value
byMM584	—		—	—	—	—		
byMM1918	Tsynth8'-sp100-pGPD-intronRAD9-yEGFPfragment-intronRAD9'-pTEF1' pMET25-Ago1 pGAL1-Dcr1-Tsynth6 Tsynth8_pCYC1noTATA-yEGFP	Mean(FI)	1854.36	1863.41	930.59	1435.34	0.0001 (***)	1.60
		SD	35.53	38.02	62.10	80.74		
		Replicates	3	5	3	5		

Table S15. Analysis of the NOT gates in Figure 9. Mean fluorescence intensity (FI) and standard deviation (SD) are expressed in arbitrary units (A.U.) and obtained from a variable number of independent experiments (replicates) that were carried out in different days. The p-value was calculated using the two-sided Welch's t-test to compare mean FIs from the (sub)circuits in the presence and absence of the galactose. Gate performance is quantified with the OFF/ON ratio.

Strains	Content (every TU, unless otherwise specified, ends with CYC1t)		NOT GATE		p-value	OFF/ON ratio
			0	1		
			1	0		
byMM584	—		—	—		
byMM254	pTEF1-Ago1<br>pTEF2-Dcr1<br>Tsynth8_pCYC1noTATA-yEGFP	Mean(FI)	1597.60	896.32	0.0011 (**)	0.56
		SD	244.52	162.69		
		Replicates	5	5		
byMM352	pGAL1-intronRAD9-yEGFPfragment-CYC1t-pGPD-(intronRAD9-yEGFPfragment)'-Tsynth8<br>pTEF1-Ago1<br>pTEF2- Dcr1<br>Tsynth8_pCYC1noTATA-yEGFP	Mean(FI)	2218.90	440.50	0.0010 (**)	0.35
		SD	286.94	33.10		
		Replicates	4	4		
byMM283	pTEF1-Ago1<br>pTEF2-Dcr1<br>Tsynth8_pCYC1noTATA-yEGFP	Mean(FI)	1673.64	1106.17	0.0022 (**)	0.66
		SD	245.01	179.44		
		Replicates	7	4		
byMM348	pGAL1-intronRAD9-yEGFPfragment-CYC1t-pGPD-(intronRAD9-yEGFPfragment)'-Tsynth8<br>pTEF1-Ago1<br>pTEF2- Dcr1<br>Tsynth8_pCYC1noTATA-yEGFP	Mean(FI)	1679.13	387.21	0.0003 (***)	0.35
		SD	139.57	10.63		
		Replicates	4	3		
byMM2018	pGAL1-intronRAD9-yEGFPfragment-intronRAD9-CYC1t-pGPD-(intronRAD9-yEGFPfragment)'-Tsynth8<br>pTEF1-Ago1<br>pTEF2- Dcr1<br>Tsynth8_pCYC1noTATA-yEGFP	Mean(FI)	1404.31	483.73	<0.0001 (****)	0.52
		SD	64.99	44.59		
		Replicates	3	3		

Table S16. All integrative plasmids employed in this study.

Plasmid	Construct
pMM433	pRSII406-pGAL1-BsaI(TTAC)-sp-BsaI(GCTT)-CYC1t (siRNA precursor acceptor vector)
pMM473	pMM433-siRNA precursor(hairpin arm:276 nt, cap:67 nt)
pMM260	pRSII405moclo-Tsynth8_pCYC1noTATA-yEGFP-CYC1t
pMM1	pRS404-pTEF1-Ago1
pMM469	pRSII403gg-pTEF2-Dicer(HIS)-CYC1t
pMM523	pRSII416-siRNA precursor(hairpin arm:276 nt, cap:67 nt)
pMM537	pRSII406-pGAL1-siRNA_precursor(276nt)-CYC1t-DEG1t'
pMM1489	pRSII406-pGPD-siRNA_precursor-Tsynth6
pMM1532	pRSII406-pGAL1-siRNA_precursor-Tsynth6
pMM1562	pRSII406-pGAL1-sirna_precursor-polyT (no terminator)
pMM1464	pRSII403-pGAL1-ATG-FLAGtag-GS-SpeI-Dcr1-XbaI-Tsynth6
pMM1609	pRSII404-pGAL1-FLAGtag-Dcr-GSG-ERBV2A-HIStag-Ago1-CYC1t(Ag22313)
pMM1596	pRSII403-pMET25-ATG-FLAGtag-GS-SpeI-Dcr1-XbaI-Tsynth6
pMM1553	pRSII403-pCUP1-ATG-FLAGtag-GS-SpeI-Dcr1-XbaI-Tsynth6
pMM1597	pRSII404-pMET25-Ago1-CYC1t (Ag22313)
pMM504	pRSII406-Tsynth8'-sp100-pGAL1-intronRAD9-yEGFPfragment-intronRAD9'-pTEF1'-CYC1t
pMM53	pRSII406-pGPD-yEGFP-CYC1t
pMM90	pRSII406-pTEF1-yEGFP-CYC1t
pMM450	Tsynth8'-sp100-pGPD-yEGFP-CYC1t
pMM451	pTEF1-yEGFP-sp100'-Tsynth8
pMM472	pRSII406-CYC1t'-pTEF1-yEGFP-spacer100'-Tsynth8
pMM481	pRSII406-Tsynth8'-spacer100-yEGFP'-pTEF1'-CYC1t
pMM483	pRSII406-Tsynth8'-spacer100-pGPD-yEGFP-pTEF1'-CYC1t
pMM492	pRSII406- Tsynth8'-sp100-pGPD-intronRAD9-yEGFPfragment-intronRAD9'-pTEF1'-CYC1t
pMM423	pRSII406-pGAL1-yEGFP-CYC1t
pMM482	pRSII406-Tsynth8'-spacer100-pGAL1-yEGFP-CYC1t
pMM500	pRSII406-Tsynth8'-sp100-pGAL1-yEGFP-pTEF1'-CYC1t
pMM511	pRSII406-Tsynth8'-sp100-pGAL1-intronRAD9-yEGFPfragment-pTEF1'-CYC1t
pMM510	pRSII406-Tsynth8'-sp100-pGPD-intronRAD9-yEGFPfragment-pTEF1'-CYC1t
pMM519	pRSII406-pGA1L-intronRAD9-yEGFPfragment-CYC1t-pGPD-(intronRAD9-yEGFPfragment)'-Tsynth8
pMM514	pRSII406-pGPD-(intronRAD9-yEGFPfragment)'-Tsynth8
pMM518	pRSII406-pGAL-intronRAD9-yEGFPfragment-CYC1t
pMM1629	pRSII406-pGAL1-RAD9-yEGFPfragment-RAD9-CYC1t-pGPD-yEGFPfragment'-RAD9'-Tsynth8
pMM1474	pRSII406-pGPD-half_hairpin-A
pMM1625	pUC57-RAD9-yEGFPfragment-RAD9-CYC1t
pMM1626	pRSII406-pGAL1-RAD9-yEGFPfragment-RAD9-CYC1t

Table S17. All yeast strains implemented in this study.

Strain	Genotype
byMM234	byMM111 pMM260::LEU2
byMM250	byMM111 pMM260::LEU2 pMM469::HIS3
byMM254	byMM111 pMM260::LEU2 pMM469::HIS3 pMM1::TRP1
byMM283	byMM111 pMM260::LEU2 pMM469::HIS3 pMM1::TRP1
byMM279	byMM111 pMM260::LEU2 pMM469::HIS3 pMM1::TRP1 pMM473::URA3
byMM280	byMM111 pMM260::LEU2 pMM469::HIS3 pMM1::TRP1 pMM473::URA3
byMM355	byMM111 pMM260::LEU2 pMM469::HIS3 pMM1::TRP1 pMM523::URA3
byMM356	byMM111 pMM260::LEU2 pMM469::HIS3 pMM1::TRP1 pMM523::URA3
byMM380	byMM111 pMM260::LEU2 pMM469::HIS3 pMM1::TRP1 pMM537::URA3
byMM1493	byMM111 pMM260::LEU2 pMM1::TRP1
byMM1849	byMM111 pMM260::LEU2 pMM1::TRP1 pMM469::HIS3
byMM1852	byMM111 pMM260::LEU2 pMM1::TRP1 pMM469::HIS3 pMM537::URA3
byMM1850	byMM111 pMM260::LEU2 pMM1::TRP1 pMM469::HIS3 pMM1532::URA3
byMM1853	byMM111 pMM260::LEU2 pMM1::TRP1 pMM469::HIS3 pMM1532::URA3
byMM1851	byMM111 pMM260::LEU2 pMM1::TRP1 pMM469::HIS3 pMM1562::URA3
byMM1854	byMM111 pMM260::LEU2 pMM1::TRP1 pMM469::HIS3 pMM1562::URA3
byMM1695	byMM111 pMM260::LEU2 pMM1::TRP1 pMM1464::HIS3
byMM1696	byMM111 pMM260::LEU2 pMM1::TRP1 pMM1464::HIS3
byMM1697	byMM111 pMM260::LEU2 pMM1::TRP1 pMM1464::HIS3 pMM1489::URA3
byMM1698	byMM111 pMM260::LEU2 pMM1::TRP1 pMM1464::HIS3 pMM1489::URA3
byMM1699	byMM111 pMM260::LEU2 pMM1::TRP1 pMM1464::HIS3 pMM1489::URA3
byMM1846	byMM111 pMM260::LEU2 pMM1::TRP1 pMM1553::HIS3
byMM1863	byMM111 pMM260::LEU2 pMM1::TRP1 pMM1553::HIS3 pMM1489::URA3
byMM1864	byMM111 pMM260::LEU2 pMM1::TRP1 pMM1553::HIS3 pMM1489::URA3
byMM1897	byMM111 pMM260::LEU2 pMM1::TRP1 pMM1596::HIS3
byMM1914	byMM111 pMM260::LEU2 pMM1::TRP1 pMM1596::HIS3 pMM1489::URA3
byMM1884	byMM111 pMM260::LEU2 pMM1489::URA3
byMM1977	byMM111 pMM260::LEU2 pMM1489::URA3 pMM1609::TRP1
byMM1892	byMM111 pMM260::LEU2 pMM1489::URA3 pMM1464::HIS3
byMM1893	byMM111 pMM260::LEU2 pMM1489::URA3 pMM1464::HIS3
byMM1900	byMM111 pMM260::LEU2 pMM1489::URA3 pMM1464::HIS3 pMM1597::TRP1
byMM1901	byMM111 pMM260::LEU2 pMM1489::URA3 pMM1464::HIS3 pMM1597::TRP1
byMM1902	byMM111 pMM260::LEU2 pMM1489::URA3 pMM1464::HIS3 pMM1597::TRP1
byMM1958	byMM111 pMM260::LEU2 pMM1597::TRP1
byMM1963	byMM111 pMM260::LEU2 pMM1597::TRP1 pMM1553::HIS3
byMM1976	byMM111 pMM260::LEU2 pMM1597::TRP1 pMM1553::HIS3 pMM1489::URA3
byMM38	byMM2 pMM90::URA3
byMM242	byMM2 pMM450::URA3
byMM243	byMM2 pMM451::URA3
byMM251	byMM2 pMM472::URA3
byMM270	byMM2 pMM481::URA3

byMM282	byMM2 pMM483::URA3
byMM103	byMM2 pMM213::URA3
byMM276	byMM111 pMM260::LEU2 pMM469::HIS3 pMM1::TRP1 pMM492::URA3
byMM277	byMM111 pMM260::LEU2 pMM469::HIS3 pMM1::TRP1 pMM492::URA3
byMM278	byMM111 pMM260::LEU2 pMM469::HIS3 pMM1::TRP1 pMM492::URA3
byMM307	byMM111 pMM260::LEU2 pMM469::HIS3 pMM1::TRP1 pMM492::URA3
byMM308	byMM111 pMM260::LEU2 pMM469::HIS3 pMM1::TRP1 pMM492::URA3
byMM224	byMM3 pMM423::URA3
byMM281	byMM2 pMM482::URA3
byMM292	byMM2 pMM500::URA3
byMM305	byMM111 pMM260::LEU2 pMM469::HIS3 pMM1::TRP1 pMM504::URA3
byMM306	byMM111 pMM260::LEU2 pMM469::HIS3 pMM1::TRP1 pMM504::URA3
byMM1911	byMM111 pMM260::LEU2 pMM492::URA3
byMM1912	byMM111 pMM260::LEU2 pMM492::URA3 pMM1::TRP1
byMM1975	byMM111 pMM260::LEU2 pMM492::URA3 pMM1::TRP1 pMM1553::HIS3
byMM1917	byMM111 pMM260::LEU2 pMM492::URA3 pMM1::TRP1 pMM1464::HIS3
byMM1913	byMM111 pMM260::LEU2 pMM492::URA3 pMM1597::TRP1
byMM1918	byMM111 pMM260::LEU2 pMM492::URA3 pMM1597::TRP1 pMM1464::HIS3
byMM1964	byMM111 pMM260::LEU2 pMM492::URA3 pMM1::TRP1 pMM1596::HIS3
byMM312	byMM111 pMM260::LEU2 pMM469::HIS3 pMM1::TRP1 pMM510::URA3
byMM313	byMM111 pMM260::LEU2 pMM469::HIS3 pMM1::TRP1 pMM510::URA3
byMM333	byMM111 pMM260::LEU2 pMM469::HIS3 pMM1::TRP1 pMM511::URA3
byMM334	byMM111 pMM260::LEU2 pMM469::HIS3 pMM1::TRP1 pMM511::URA3
byMM352	byMM111 pMM260::LEU2 pMM469::HIS3 pMM1::TRP1 pMM519::URA3
byMM348	byMM111 pMM260::LEU2 pMM469::HIS3 pMM1::TRP1 pMM519::URA3
byMM2018	byMM111 pMM260::LEU2 pMM469::HIS3 pMM1::TRP1 pMM1629::URA3

Table S18. DNA sequences used in this work.

DNA fragments	sequence
pGAL1	ATATACATATCCATATCTAATCTTACTTATATGTTGTGGAAATGTAAA<br>GAGCCCCATTATCTTAGCCTAAAAAAACCTTCTCTTTGGAACTTTCA<br>GTAATACGCTTAACTGCTCATTGCTATATTGAAGTACGGATTAGAAG<br>CCGCCGAGCGGGTGACAGCCCTCCGAAGGAAGACTCTCCTCCGTGC<br>GTCCTCGTCTTCACCGGTCGCGTTCCTGAAACGCAGATGTGCCTCGC<br>GCCGCACTGCTCCGAACAATAAAGATTCTACAATACTAGCTTTTATG<br>GTTATGAAGAGGAAAAATTGGCAGTAACCTGGCCCCACAAACCTTC<br>AAATGAACGAATCAAATTAACAACCATAGGATGATAATGCGATTAG<br>TTTTTTAGCCTTATTTCTGGGGTAATTAATCAGCGAAGCGATGATTT<br>TTGATCTATTAACAGATATATAAATGCAAAAACTGCATAACCACTTT<br>AACTAATACTTTCAACATTTTCGGTTTGTATTACTTCTTATTCAAATG<br>TAATAAAAGTATCAACAAAAAATTGTTAATATACCTCTATACTTTAA<br>CGTCAAGGAGAAAAAACtata
pCUP1	CTAGTTAGAAAAAGACATTTTTGCTGTCAGTCACTGTCAAGAGATTC<br>TTTTGCTGGCATTTCTTCTAGAAGCAAAAAGAGCGATGCGTCTTTTC<br>CGCTGAACCGTTCCAGCAAAAAAGACTACCAACGCAATATGGATTG<br>TCAGAATCATATAAAAGAGAAGCAAATAACTCCTTGTCTTGTATCAA<br>TTGCATTATAATATCTTCTTGTTAGTGCAATATCATATAGAAGTCATC<br>GAAATAGATATTAAGAAAAACAAACTGTACAATCAATCAATCAATC<br>ATCACATAAA
pMET25	CTTCGGATGCAAGGGTTCGAATCCCTTAGCTCTCATTATTTTTTGCTT<br>TTTCTCTTGAGGTCACATGATCGCAAAATGGCAAATGGCACGTGAA<br>GCTGTCGATATTGGGGAACTGTGGTGGTTGGCAAATGACTAATTAA<br>GTTAGTCAAGGCGCCATCCTCATGAAAACTGTGTAACATAATAACC<br>GAAGTGTCGAAAAGGTGGCACCTTGTCCAATTGAACACGCTCGATG<br>AAAAAAATAAGATATATATAAGGTTAAGTAAAGCGTCTGTTAGAAA<br>GGAAGTTTTTCCTTTTTCTTGCTCTCTTGTCTTTTCATCTACTATTTCC<br>TTCGTGTAATACAGGGTCGTCAGATACATAGATACAATTCTATTACC<br>CCCATCCATAC
pGPD	cagttcgagtttatcattatcaatactgccatttcaaagaatacgtaaataattaatagta<br>gtgattttcctaactttatttagtcaaaaaattagccttttaattctgctgtaacccgtacat<br>gcccaaaatagggggcgggttacacagaatatataacatcgtaggtgtctgggtgaaca<br>gtttattcctggcatccactaaatataatggagcccgctttttaagctggcatccagaaaa<br>aaaaagaatcccagcaccaaaatattgttttcttcaccaaccatcagttcataggtccatt<br>ctcttagcgcaactacagagaacaggggcacaaacaggcaaaaaacgggcacaacctc<br>aatggagtgatgcaacctgcctggagtaaatgatgacacaaggcaattgacccacgcat<br>gtatctatctcattttcttacaccttctattaccttctgctctctctgatttggaaaaagctga<br>aaaaaaaggttgaaaccagttccctgaaattattcccctacttgactaataagtatataa<br>agacggtaggtattgattgtaattctgtaaatctatttcttaaacttcttaaattctactttta<br>tagttagtcttttttttagttttaaaacaccaagaacttagtttcgaataaacacacataaa<br>caaacaaa
CYC1t_ATC	CATGTAATTAGTTATGTCACGCTTACATTCACGCCCTCCCCCCACATC<br>CGCTCTAACCGAAAAGGAAGGAGTTAGACAACCTGAAGTCTAGGTC<br>CCTATTTATTTTTTTATAGTTATGTTAGTATTAAGAACGTTATTTATAT<br>TTCAAATTTTTCTTTTTTTTCTGTACAGACGCGTGTACGCATGTAACA<br>TTATACTGAAAACCTTGCTTGAGAAGGTTTTGGGACGCTCGAAGGC<br>TTTAATTTGCAAGCTatc
Tsynth6	TATATATTTAATAAAGAGTATCATCTTTCAAA

Tsynth8	TATATAAACTCATTTACTTATGTAGGAATAAAGAGTATCATCTTTCA<br>AA
DEG1t'	AATAATATATAAACCTGTATAATATAACCTTGAAGACTATATTTCTTT<br>TC
Poly (T)	CTCGAGTTTTTTTTTTTTTTTTTTTTTTTTTTTTTTTTTTTTTTTTTTTTTTTT<br>TTTTTTTTTTTTTTTTTTTTTTTTTTTTTTTTTTTTTTTTTTTTTTTTTTTT<br>TTTTTTTTTTTTTTTTTTTTTTTTTTTTTTTTTTTTTTTTTTTTTTTTTTTT<br>TTTTTTTTTTTTTTTTTTTTTTTTTTTTTTTTTTTTTTTTTTTTTTTTTTTT<br>TTTTTTTTTTTTTTTTTTTTTTTTTTTTTTTTTTTTTTTTTTTTTGAGCTC
pTEF2	GCTACCTATATTCCACCATAACATCAATCATGCGGTTGCTGGTGTAT<br>TTACCAATAATGTTTAATGTATATATATATATATATGGGGCCGTATA<br>CTTACATATAGTAGATGTCAAGCGTAGGCGCTTCCCCTGCCGGCTGT<br>GAGGGCGCCATAACCAAGGTATCTATAGACCGCCAATCAGCAAACT<br>ACCTCCGTACATTCATGTTGCACCCACACATTTATACACCCAGACCG<br>CGACAAATTACCCATAAGGTTGTTTGTGACGGCGTCGTACAAGAGA<br>ACGTGGGAACTTTTTAGGCTCACCAAAAAAGAAAGAAAAAATACGA<br>GTTGCTGACAGAAGCCTCAAGAAAAAAAAAAATTCTTCTTCGACTAT<br>GCTGGAGGCAGAGATGATCGAGCCGGTAGTTAACTATATATAGCTA<br>AATTGGTTCCATCACCTTCTTTTCTGGTGTCGCTCCTTCTAGTGCTAT<br>TTCTGGCTTTTCCTATTTTTTTTTTTCCATTTTTCTTTCTCTCTTTCTAAT<br>ATATAAATTCTCTTGCATTTTCTATTTTTCTCTCTATCTATTCTACTTGT<br>TTATTCCCTTCAAGGTTTTTTTTTAAGGAGTACTTGTTTTTAGAA<br>TATACGGTCAACGAACTATAATTAACTAAAC
pTEF1	ATAGCTTCAAAATGTTTCTACTCCTTTTTTACTCTTCCAGATTTTCTCG<br>GACTCCGCGCATCGCCGTACCACTTCAAAACACCCAAGCACAGCAT<br>ACTAAATTTCCCCTCTTTCTTCCTCTAGGGTGTCGTTAATTACCCGTA<br>CTAAAGGTTTGGAAAAGAAAAAAGAGACCGCCTCGTTTCTTTTTCTT<br>CGTCGAAAAAGGCAATAAAAATTTTTATCACGTTTCTTTTTCTTGAA<br>AATTTTTTTTTTGATTTTTTTCTCTTTCGATGACCTCCCATTGATATTT<br>AAGTTAATAAACGGTCTTCAATTTCTCAAGTTTCAGTT<br>TCATTTTTCTTGTTCTATTACAACTTTTTTTACTTCTTGCTCATTAGAA<br>AGAAAGCATAGCAATCTAATCTAAGTTT
pTEF1’	AAACTTAGATTAGATTGCTATGCTTTCTTTCTAATGAGCAAGAAGTA<br>AAAAAAGTTGTAATAGAACAAGAAAAATGAAACTGAAACTTGAGA<br>AATTGAAGACCGTTTATTAACTTAAATATCAATGGGAGGTCATCGA<br>AAGAGAAAAAAATCAAAAAAAAAATTTTCAAGAAAAAGAAACGTG<br>ATAAAAATTTTTATTGCCTTTTTCGACGAAGAAAAAGAAACGAGGC<br>GGTCTCTTTTTTCTTTTCCAAACCTTTAGTACGGGTAATTAACGACAC<br>CCTAGAGGAAGAAAGAGGGGAAATTTAGTATGCTGTGCTTGGGTG<br>TTTTGAAGTGGTACGGCGATGCGCGGAGTCCGAGAAAATCTGGAA<br>GAGTAAAAAAGGAGTAGAAACATTTTGAAGCTAT
siRNA precursor	GGATCCCCAAAGATGACGGTAACTACAAGACCAGAGCTGAAGTCA<br>AGTTTGAAGGTGATACCTTAGTTAATAGAATCGAATTAAAAGGTAT<br>TGATTTTAAAGAAGATGGTAACATTTTAGGTCACAAATTGGAATAC<br>AACTATAACTCTCACAATGTTTACATCATGGCTGACAAACAAAAGAA<br>TGGTATCAAAGTTAACTTCAAAATTAGACACAACATTGAAGATGGTT<br>CTGTTCAATTAGCTGACCATTATCAACAAAATACTCCAATTGGTGAT<br>GGTCCAGTCTTGTTACCAGACAACCTCTATCTTTACCAATTAGTTTCA<br>ATGTTTAGTAAGTCTAGAGTTTGAAAAAAGTTCCAACACACCTGATT<br>TTGTTGTCTGGTAACAAGACTGGACCATCACCAATTGGAGTATTTTG<br>TTGATAATGGTCAGCTAATTGAACAGAACCATCTTCAATGTTGTGTC

	TAATTTTGAAGTTAACTTTGATACCATTCTTTTGTTTGTCAGCCATGA<br>TGTAAACATTGTGAGAGTTATAGTTGTATTCCAATTTGTGACCTAAA<br>ATGTTACCATCTTCTTTAAAATCAATACCTTTTAATTCGATTCTATTAA<br>CTAAGGTATCACCTTCAAACTTGACTTCAGCTCTGGTCTTATTACTTA<br>TCCACTCAATCGCTTCTCGAG
Ago1	ATGTCATCCAATTCGGAGGAGAACAGTCAAGTTCCTCCCCTTGATGC<br>CACCGCTGCTGCAACTAAACCAAAAAAGGCCAAGAAACCAAAGGTT<br>AAGAAGCCAAAGGATTCCGCTGAGGCATCTTCATCACCAGCTGCTG<br>AGGGCACTGCTGAAGCCAAGCCAAAAAAGGCTAAGAAATCAAAGA<br>CTAAGAAGTCAAAGGAGTCTGCCGAGGTATCGCCAGCTCCAGCTGA<br>TGAAACTACTTCTGCTGGTGTAGATGCTAAACCTAAGAAGGCTAAA<br>AAGTCAAAGGTCAAGAAGCCAAAGGATTCTACTGAATCATCTCCTG<br>CTCCAAGTAATGAACCTCCAGCCGCCGAAGTTGCCGCCGAAGATGC<br>AAAGTCTAAAAAGGTTAAGAAACCAAAGGCCAAGAAACCAAAGGA<br>ATCTACTGAATCATCTCCTGCTCCAGGTCAGGAAGCCGCTGCTACTG<br>AAGGTGCCACAGAAGATAAACCTAAGAAGGTTAAGAAATCAAAGG<br>CTAAGAAGGCAAAGGAATCTGTTGAATCATCTCCTGCCGCCACTGA<br>ATCTGTCTCTGAGAAGACTGCAAAGAAATCTAAGAAGCCAAAGGCT<br>AAGAAATCTACTTCTCCTGAAACAACTGAAGAAATAACTGAAGAAT<br>CTACCGAATCAAAGGAAAAGAAGACAAAGACAAAGAAACCAAAGG<br>AAAAGAAGTCATCTCCATCTACTGCCACTTCGACTGCTGCTTCAAAA<br>CCAGTTACTTCCATTGCGGGAGTCACCATTCCAGGCAAGACTTTTGA<br>TTTGACAGATGTTCCTCCATCTAAACCTGCTCCAAAGATGGTCCCGG<br>AAGCCTATAAATTACAAACTAGAGTGGATTACGGTACTAAGGGTAC<br>CAAAGTGGACGTCTTGACTAATCATATACTACTATCTGTTGGTGATG<br>ATGTCCCTCAAGATGAAAGAGCCTCTCAATTGGATCCATGGTGGAA<br>ATCTGCATTTGTATATACCTATAACATTACTTTTGCCGTTCCACAGAG<br>TAATTCACCACGTAAAGGTCCAGCGCCTGCTTTATCCAAACCAAAGA<br>AATACGAATTGGTAGAATCTTTGTTTCACGAGGATGAAACCTTGTTC<br>AAATATAAAGATCGTATCTCCTTCAATGGTGAAGATACTCTATACTC<br>TCATGTCCCATTGGAGGAATTCACTTTATTTGATGGTTGTTGGGATG<br>TCAGTAACAAGCAAAAGAAAAGAAGACAAGAAGTTGTTGGTCTCA<br>ACAGTAGAGCCAAGGAAATTAATGACTTGGCTGCTCAAGTTACTTT<br>AAAATTTGCTGATAAAGTCCCATTGGGTGATATTTATAAGGCTACCA<br>CTTCAAAGGATCCAGAAGAACAAGAAAATAAGATGGCTAACGCTG<br>ATAAGGTTGCTTTATTGTCTTTGATGGGTGTCAAATTCTTAAACACA<br>AAGGAACAAATCTTCCAACTTAACGGTAATAAATTCTTTATTTTTAAT<br>GAACACGCAATTGCTACTCCATTCCAAATTGGTGGGTTCTTAATGCA<br>TGGGTTCACAGTTTCCCTACGATATGCTTACGGGTCCGTTTTGTTAA<br>ATACTGTTAACGTCTGCCTCCCATTTGTTAAGTGGACTAAATATTTAC<br>CAGGTGATGCTAAATTCAAGGAAAATGAAAAGACTCAATACAGTTT<br>GTTAGATTGGATTATTGAATGTATGCACCAGGCCAGTGCTCAAAGA<br>GGTCAAAAGCTAAGAGGCCCTCCATCTGCCAAGGATATCAATTTTTT<br>CATTGACAAGAATAGAGACATTAAAGATCTATTGAAGGGTTTGAAA<br>TGTTACAGACCATACATTAATTATTCTGTTAACCCAGATGGAACTCC<br>AAAACCACCAAAAAAGATGCAAGCTAAGGGTATCGTTGGTTTCGTA<br>AGAGAGACTCCAGATTCTATGAAATTCAGAACTTTACCAAGTAACAT<br>GGAAAAGAATGGTGTACCAAAACCAGGTGAAAAGGAAATCATGGT<br>CACAACAACCGCCTACTTTGCCAAGAAGTATGACATCAAATTGAAAT<br>ACCCAGATGTTAAAATGGTGAGTCTAGGTGGTTCGAATGTTGTCCC<br>AGCTGAATGTTTAACCATTGTTCCAGGTCAGAAATTAAAGGGTTTG

Dcr1	GTTTATGACGAAAAAGCCGTTATTGATTTCACTGCTTTAAGACCAAG<br>TGAAAAATTCAGAGCCATCACCAACTTGGCATTACCTGCCATTAAGA<br>GAGCTTTATCAACTGAAGAAGAAAATGCTAAGGCTCCACATGATTC<br>TGGTTACACTTTCATGAAGGTCCCATCTCGTGTCATTGACGCTCCTG<br>TGGTTCAATTCAAGAACACTACAGTCACATATGTGGATAAACCATTT<br>GGTACTAAGAATGGTAAGAATAATCATGAAGAAACTAAGGGTAATT<br>GGAATTTAAAGGATCATAAATTCATTACTGTTCCAAAGGAACCAAT<br>GCACTTGAGAGCTATCTTTATTAATGATTCTGATAAGTCTCCACCAG<br>TTTCTGTCATGGATGAATTGAAGGCCTCTTTGAGCAAATTCGCTGAA<br>GATGTCGCTGATGTCGGTGTTAACTTTGACGTATCCATGGCCCCAAT<br>TTTAATTAACAATTTTAATGCCCCAATAAAGAAGGTTACTGGTGGCT<br>TTGGTGGTAGAGGTGGACGTGGTGGTAGAGGCGGCCGTGGTGGC<br>CGTGGTGGTAGAGGAGGTCGTGGTGGTTTCGGCGGTGGCCGTGGT<br>GAAACTACTTATGAATTAACTCCAGGTGAAGAAAAACTACGTCACTT<br>GTTAGCCAACGTCCCAGAAAAGACTTATGTCTTATTCGTCTTGGGTC<br>GTGGGGATGACTCTGCTATTTACAACAGATTGAAATATTTAGCTGAT<br>TTGACGTACGGTGTCATTAACAATTGTGTTATTTGGAACAAGTTCAG<br>AAAGTGTTCCACTCAATACAATGTTAATGTGGTAATGAAGATGAAC<br>TTGAAGTTAGAAGGTGCTAACCACTCCTTATGTGCAGAAGATATAA<br>ACTTACTGAAGGATGAAAAATCAGGATTACCATTCATGATTTTAGGT<br>GCTGATGTTACACATTATCCAGAAAAGGATCAGAATTCTATCTCTGC<br>ATTGGTGGGTTCTTTTGATGACAAATTTGCTCAATTCCCAGGTTCAT<br>ATATGTTACAAAGTGGTCCAGGTGAAGAAATAATTGCTGGTATCGG<br>TAACATGGTTCTACAGAGATTGAAACTGTATCAAAAGCACAACAAT<br>GGCAAACTACCTCCAAAGATTCTATTCTACAGAGATGGTGTTTCTGA<br>ATCCCAATTCTCACAAATTGTTCAAATTGAAGTTAAGGGTTTGAAAC<br>AAGCATTGAAGAAATTCGGTAGTGAATTAAACAAGGGTGTCAACTA<br>TAACCCATCTGTCACCACAATTTGTGTTGTCAAGAGAAATCAAATTA<br>GATTTATGCCACTTGAGCAAAATGCTATAAATGAAAAGGGTGAAGT<br>TGCTGCCGTTCAATCATTCGAGAACGTTATGCCAGGTACTGTTGTTG<br>ATCGTGGTATCACCTCTAGTGCTCACTTCGATTTCTTCTTGCAATCTC<br>AACAACCATTGAAGGGTACTGGTGTTCCATGTCATTACTGGTGTATC<br>TACGACGAAAATCAATTCAATTCTGATTACTTGCAACAAGTTACTCA<br>CGCTTTGTGTTACTTATTCGGTAGATCGAGTACAAGTATTAAGGTCG<br>CATCTCCTGTTTATTACGCTGATTTATTGTGTGAACGTGGTGCTGCA<br>TTCTTCAAGGCTAACTTTGAGCTTGCTCAATACGAATTTTCTAAGGA<br>GAGGAAGAACAGAGATGACGTTATACCGACCGGTAAATTACTACA<br>ACCTGTTCATAAGAATGTCACTGACATCATGTACTACATATGA<br>AATAGAGAAAAAAGCGCCGATCTAAGTAAAACATCAGATGCTCCAT<br>ACAATGAACTCGATGCCAAAAATTTAAGAAATTTCTATAAGGTTCAA<br>AATGCATGTGCGCAATTGAGGGAATCCATAAAAGTAATTTACGAAA<br>ACGGGTTATCATCCGATCAATTGAATACTATGGCGAAACATGGGAA<br>CGACCTAGAAAAATCTATTGCAAACAGTCCTGCTATGTCCGTAGCAA<br>GTTGCTTGAACCAAGTTAGACCCACCCTAGACATCAAGAATATCTTT<br>GATCATTATAAATTTGAAAATAATTCATCACCAGTTGATCCTTATGTC<br>CATTATCCCGTATGTTCTGATCAAAATTTGGAAAATTTGGCTTTCATT<br>CATAGATCTTTGCCGAATATGAATGTCAAATTGACTGAATTGCAAAA<br>GACCGTAATGAGTAATGAACGTTTGGAATTTTTAGGTGATAGTTGG<br>CTTGGCGCCTTAGTGGCGTATATTATTTATAAGAAATATCCTTATGC<br>TAATGAAGGTGCCTTATCAAAGATGAAGGAAGCTATCGTTAACAAC<br>AACAATTTGGAAAAGATCTGTGAAAAACTTGGCTTTAAAGAAAGAT

	TAAAGGAAAATATTCCACGTTCTTCAATGAAAATCAAAGATAGATTA ACCAAGAATTACGCAGACTGTGTGGAAGCCTACATTGGCGCATTGG TTATTGATAGATTTTCAACAGAATTTAATGATGTTGCTCTTTGGCTTG AAGAACTATCTGAGGAACATTTCATTGAACTAGGACCTATGATGGT CAAAGAGCCTTTAAACAAAAATGCAAAGGGTGAACTAGGTGCGTTC TTACAATTTAATAATATTGGAGCCAAAATATCATATAAAAGATTGAA TGATAAATCTCCGTTTAAGGTAGAAGTAAGATTGGGGAATAATTTG TTAGGTATTGGAGATGGTTCAAATGTGAGAGAAGCTGAACAGAGA GCCGCTATGGAAGCACTTGCTCAACCGAAATTGATTCAAAAATATTC TCTACATGATATTGAACTAGAAAGAGGCATGATTGAAGAAGCAGAT AATGTTCCCCAACTACTCAAAAGTGCGGAAATTCCCAATCAAACACC ACAAAGTCATCATTCTATCACAGAAGAAATTGATGAAGAAGAAGGT CTTACATTCCCCCATTCGAATCCATTTTTGAAAGGTACACCACCAGC AGTTTTACCTGAAATTGTAACTAATCAAATTCCACAGGCTTCTGCGG ATCATCAAGAACAAAATGTGCCTGACACTGATAAAATTGTGAATGA TGTTATGGAACGTATGAGTAAGATACTGTCGGTGATGGTTTCTGAG GCTGTTTCAAATGCACTTGGTAGACCAGCTCCAAAAAATGCACCTAT CCCTATCCCAGTAACAACCCCAGTCTCACCGCTAGCAACATCCCCAG TCTCAGTCTCAGTCCCAGCGCCAGCTCCAGCCGTAGCTGTCACCCCA TCAGTTGTGAATAATCCACCACAACCCAAAATGGTCACACAACCGG TAAGCACAAACCTAAATAAAACGCCTGAAGTTGCACTTTTAAATTCT GTGTATGATAAAGAAGCCTCAGGGAGACTTTATGCATTGTTAGGTA AATATAAATTGTATCCAGAATATAATACCGAGCAACTAGGAATGAC AGATTTTTATACGGTATGCTTTATAAAGGGTGTTGGTGTAGAAATTG GTAAAGGCCATGGTAGAAGTAAAAAGATATCCCAACATAAATCTGC AGAAGATGCTTTGAATGGAAAAGCACTGAAGGAATACTTGAGGCA TTGCAACAATCTG
HIS_tag	AGCCATCATCATCATCATCACAGC
FLAG_tag	GACTACAAAGACGATGACGACAAG
HA_tag	tacccatacgacgtcccagactacgct
GSG-ERBV2A	GGCTCGGGCGGTGCCACAAATTTTTCTTTGTTGAAGTTAGCAGGGG ATGTTGAACTTAACCCCGGCCCA
Intron RAD9	GTGTGTTGGAACTTTTTTCAAACCTTACTAAACATTGAAACTAATTG GTAAAG
Intron RAD9’	CTTTACCAATTAGTTTCAATGTTTAGTAAGGTTTGAAAAAAGTTCCA ACACAC
yEGFP fragment	ATGTCTAAAGGTGAAGAATTATTCACTGGTGTTGTCCCAATTTTGGT TGAATTAGATGGTGATGTTAATGGTCACAAATTTTCTGTCTCCGGTG AAGGTGAAGGTGATGCTACTTACGGTAAATTGACCTTAAAATTTATT TGTACTACTGGTAAATTGCCAGTTCCATGGCCAACCTTAGTCACTAC TTTCGGTTATGG
yEGFP	ATGTCTAAAGGTGAAGAATTATTCACTGGTGTTGTCCCAATTTTGGT TGAATTAGATGGTGATGTTAATGGTCACAAATTTTCTGTCTCCGGTG AAGGTGAAGGTGATGCTACTTACGGTAAATTGACCTTAAAATTTATT TGTACTACTGGTAAATTGCCAGTTCCATGGCCAACCTTAGTCACTAC TTTCGGTTATGGTGTTCAATGTTTTGCGAGATACCCAGATCATATGA AACAACATGACTTTTTCAAGTCTGCCATGCCAGAAGGTTATGTTCAA GAAAGAACTATTTTTTTCAAAGATGACGGTAACTACAAGACCAGAG CTGAAGTCAAGTTTGAAGGTGATACCTTAGTTAATAGAATCGAATT AAAAGGTATTGATTTTAAAGAAGATGGTAACATTTTAGGTCACAAA

	TTGGAATACAACTATAACTCTCACAATGTTTACATCATGGCTGACAAACAAAAGAATGGTATCAAAGTTAACTTCAAAATTAGACACAACATTGAAGATGGTTCTGTTCAATTAGCTGACCATTATCAACAAAATACTCCAATTGGTGATGGTCCAGTCTTGTTACCAGACAACCATTACTTATCCACTCAATCTGCCTTATCCAAAGATCCAAACGAAAAGAGgGACCACATGGTCTTGTTAGAATTTGTTACTGCTGCTGGTATTACCCATGGTATGGATGAATTGTACAAATAA
CYC1t	CATGTAATTAGTTATGTCACGCTTACATTCACGCCCTCCCCCCACATCCGCTCTAACCGAAAAGGAAGGAGTTAGACAACCTGAAGTCTAGGTCCCTATTTATTTTTTTATAGTTATGTTAGTATTAAGAACGTTATTTATATTTCAAATTTTTCTTTTTTTTCTGTACAGACGCGTGTACGCATGTAACATTATACTGAAAACCTTGCTTGAGAAGGTTTTGGGACGCTCGAAGGCTTTAATTTGCAAGCT
CYC1t (Ag22313)	ATTAGTTATGTCACGCTTACATTCACGCCCTCCCCCCACATCCGCTCTAACCGAAAAGGAAGGAGTTAGACAACCTGAAGTCTAGGTCCCTATTTATTTTTTTATAGTTATGTTAGTATTAAGAACGTTATTTATATTTCAAATTTTTCTTTTTTTTCTGTACAGACGCGTGTACGCATGTAACATTATACTGAAAACCTTGCTTGAGAAGGTTTTGGGACGCTCGAAGGCTTTAATTTG

Table S19. qPCR primers used in this work.

**Name**	**Primer sequences**
ot27	5’-CGTCTGGATTGGTGGTTCTATC-3’
ot28	5’-GGACCACTTTCGTCGTATTCTTG-3’
ot29	5’-GGTGTTGTCCCAATTTTGGTTG-3’
ot30	5’-GACTAAGGTTGGCCATGGAA-3’
